# Excess muscle plasma membrane leak disrupts ECM content and shifts macrophage-mediated muscle repair

**DOI:** 10.1172/jci.insight.205137

**Published:** 2026-06-23

**Authors:** GaHyun Lee, Alexander J. Fitt, Ashlee M. Long, Lauren A. Vaught, Dorothy DeBiasse, Alexander R. Keeble, Jason M. Kwon, Patrick G.T. Page, Marie-Therese Daher, Michele Hadhazy, Alexander B. Willis, David Ceja Galindo, Maxwell McCabe, Connor Lantz, Kirk C. Hansen, Rachelle H. Crosbie, Edward B. Thorp, Alexis R. Demonbreun, Elizabeth M. McNally

**Affiliations:** 1 Center for Genetic Medicine, Northwestern University Feinberg School of Medicine, Chicago, Illinois, USA.; 2 Department of Biochemistry and Molecular Genetics, University of Colorado Anschutz Medical Campus, Aurora, Colorado, USA.; 3 Department of Pathology, and; 4 Comprehensive Transplant Center, Northwestern University Feinberg School of Medicine, Chicago, Illinois, USA.; 5 Department of Integrative Biology and Physiology, and; 6 Department of Neurology, David Geffen School of Medicine, UCLA, Los Angeles, California, USA.; 7 Department of Pediatrics, Northwestern University Feinberg School of Medicine, Chicago, Illinois, USA.; 8 Heart Center, Stanley Manne Children’s Research Institute, Ann & Robert Lurie Children’s Hospital, Chicago, Illinois, USA.; 9 Department of Pharmacology, Northwestern University Feinberg School of Medicine, Chicago, Illinois, USA.

**Keywords:** Inflammation, Muscle biology, Macrophages

## Abstract

Plasma membrane repair is critical for tissue integrity, especially for elongated contractile muscle cells. Genetically mediated defects in plasma membrane resealing produce persistent leak, leading to a disordered extracellular matrix (ECM). Loss of the membrane repair protein dysferlin slows sarcolemmal resealing and promotes excess leak. Annexin A6 is also implicated in sarcolemmal repair, forming repair caps at the site of membrane disruption. On its own, deletion of the gene for annexin A6, *Anxa6*, had little effect on muscle health. In contrast, combined loss of dysferlin and annexin A6 (*DysfA6*) generated muscle fibers with profoundly defective membrane leak. Strikingly, *Anxa6* deletion in the context of loss of dystrophin (*mdxA6*) did not exacerbate muscle defects. The persistent membrane leak in *DysfA6* muscle resulted in marked macrophage infiltration with disordered macrophage polarization. Injured muscle fibers were targets of macrophage efferocytosis. Loss of *Anxa6* was associated with increased expression of annexins A1 and A2, both of which were heavily deposited into the ECM. In vitro, macrophages exposed to annexins A1 and A2 increased *Csf1* expression, consistent with a model where excess leak results in annexins A1 and A2 in the ECM, where this protein composition influences macrophage proliferation and efferocytosis.

## Abstract 2

Muscle lacking dysferlin and annexin A6, two proteins critical for muscle resealing, cause leaky membranges and invite macrophages to come take a bite.

## Graphical abstract

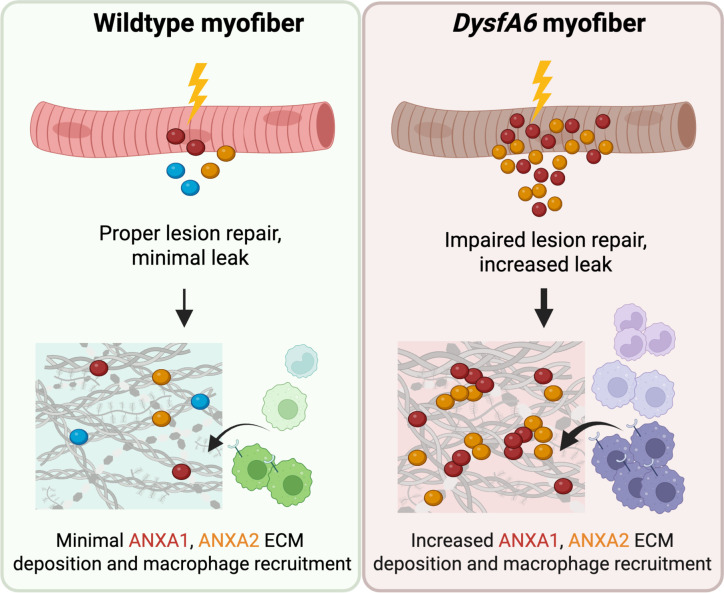

## Introduction

### Plasma membrane repair and disease.

Maintaining integrity of the plasma membrane requires robust resealing and repair. Cell types that repeatedly face mechanical stress, like skeletal muscle fibers, cardiomyocytes, and epithelial cells, are susceptible to plasma membrane disruption (1). To maintain normal tissue health and function, cells have evolved a conserved, rapid, and efficient mechanism of membrane repair. This process relies on calcium-dependent signaling and a coordinated recruitment of repair machinery, including annexins and dysferlin, to reseal the membrane (2). Failure to efficiently repair membrane lesions can result in cell death, inflammation, and chronic tissue degeneration (1).

In skeletal muscle, genetic defects affecting the sarcolemma produce muscular dystrophy, associated with distinct mechanisms. Loss of membrane-stabilizing proteins, such as dystrophin and sarcoglycan proteins, destabilize the muscle membrane, increasing susceptibility to damage, while the membrane repair machinery remains functionally intact (3, 4). In contrast, loss-of-function mutations in the membrane resealing protein dysferlin result in defective membrane repair and prolonged leak despite a stable membrane (5, 6).

### Annexins and dysferlin contribute to the resealing machinery.

Annexins are calcium-sensitive, membrane-binding proteins involved in membrane repair. With plasma membrane disruption, annexins translocate to the membrane lesion and induce membrane folding at and around the site of damage to facilitate resealing (7, 8). *Anxa6*, the gene that encodes annexin A6 (ANXA6), was identified as a genetic modifier of muscular dystrophy (9). Within seconds following membrane injury, ANXA6 translocates to the site of membrane injury and forms a repair cap that acts as a molecular “band-aid” over the lesion, a mechanism conserved across different cell types (10–12). ANXA6 facilitates the prompt recruitment of other annexins, including annexin A1 (ANXA1) and annexin A2 (ANXA2), to the repair cap at the site of membrane rupture (13, 14). Despite its role in membrane repair, removal of ANXA2 in dysferlin-deficient (*Dysf*) muscle paradoxically reduced inflammation and fat deposition in muscle and improved muscle function (15). Dysferlin localizes adjacent to the annexin protein cap at the site of membrane injury and promotes phosphatidylserine (PS) accumulation at the lesion, a key step in the repair process that engages macrophages (11, 13, 16, 17). Annexins interact with dysferlin but not with dystrophin (16), and skeletal muscle–specific expression of dysferlin is sufficient to resolve the inflammatory response in *Dysf* mice (18).

### Macrophage function in muscle injury and repair.

With progressive membrane leak, there is extrusion of cellular and membrane content into the extracellular matrix (ECM), which attracts macrophages. Macrophages are essential for tissues to recover from injury, and skeletal muscle similarly relies on macrophage function for normal recovery from injury (19). In skeletal muscle, macrophages are needed for efficient clearance of damaged myofibers, which also promotes myogenesis (20). Live imaging of myofiber injury in zebrafish demonstrates macrophage recruitment specifically to externalized PS, followed by active ingestion of annexin-rich repair caps (17). Within tissues, macrophages participate in phagocytosis, or efferocytosis, to remove cellular debris, and their antiinflammatory properties can promote healing (21–23). The TAM receptors, TYRO3, AXL, and MERTK, are receptor tyrosine kinases necessary for effective phagocytosis of injured or dying cells (24, 25). TAM receptors are involved in recognition of externalized PS, which is mediated by the activation of caspase-3 (CASP3) (26) on the target cell membrane, thereby promoting the clearance of damaged cells (21, 27). MERTK’s phagocytic and pro-resolution properties have been reported in cardiac, liver, and lung injury models (28–30). Deletion of *Mertk* impairs skeletal muscle regeneration after injury (31).

Recent studies have characterized muscle macrophage heterogeneity in acute muscle injury as well as in dystrophin-deficient mice (32–37). Key differences have been noted in the macrophage populations in acutely injured muscle compared with the chronic, concomitant degeneration and regeneration ongoing in dystrophic muscle (33–36, 38). For dystrophic muscle, these studies have primarily concentrated on dystrophin-deficient or related models of defective sarcolemma-ECM connections, in which the dominant macrophage cell type is characterized by high expression of *Spp1* and *Lgals3*, encoding the ECM proteins osteopontin and galectin-3, respectively (33, 38).

Here, we assessed the role of membrane leak and defined how sarcolemmal leak shifts ECM composition and macrophage diversity. We generated mice lacking both dysferlin (*Dysf* mice) and ANXA6 (*DysfA6* mice) to create sarcolemma that has excessively prolonged membrane resealing, and we found this resulted in a distinct ECM composition and macrophage profile. Instead of *Spp1^+^Lgals3^+^* macrophages, the macrophage population was dominated by *Mertk^+^Trem2^+^* macrophages. Leaky muscle fibers triggered enhanced macrophage efferocytosis in vivo and in vitro. ANXA1 and ANXA2 became excessively deposited in the ECM, and macrophages exposed to ANXA1 and ANXA2 expressed *Csf1*, consistent with enhanced proliferation. Together, these findings show that defective membrane repair results in an altered, pathological ECM that leads to excessive macrophage recruitment, proliferation, and phagocytosis, associated with intensified pathology.

## Results

### Loss of ANXA6 worsens muscle histopathology and function of Dysf mice.

**Figure 1:**
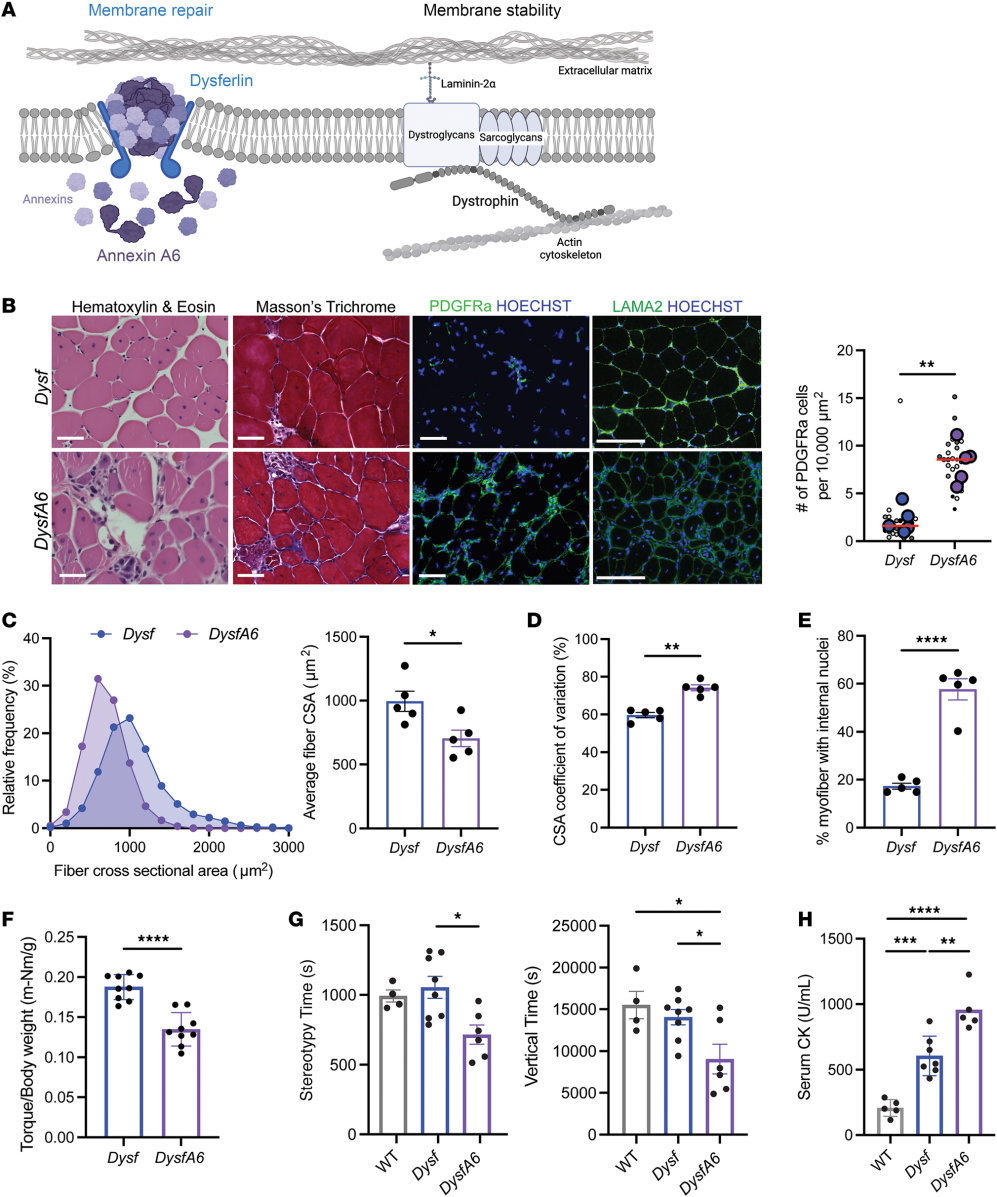
Ablation of the 2 membrane repair proteins, ANXA6 and dysferlin (*DysfA6*), intensifies dystrophic histopathology and reduces activity compared with *Dysf* alone. (**A**) Ablation of ANXA6 in addition to dysferlin (*DysfA6*) delays repair and prolongs the membrane leak compared with *Dysf*. (**B**) Representative images of quadriceps muscle from *Dysf* and *DysfA6* mice are shown, with *DysfA6* muscle exhibiting greater variation in myofiber size, increased mononuclear cell infiltrate, and fibrosis, confirmed by PDGFRα and LAMA2 staining and quantification of PDGFRα^+^ cells per 10,000 μm^2^. Small circles depict counts from each field of view (*n* = 5 mice per genotype, 5 images across 2 sections per mouse), and large circles depict average per mouse. Statistical testing compared the averages per animal. (**C**) Myofiber cross-sectional area (CSA) distribution and average confirmed *DysfA6* muscles had reduced myofiber size with greater numbers of smaller myofibers than *Dysf* muscle. (**D**) *DysfA6* muscle had increased variability of myofibers and (**E**) increased percentage of myofibers with internal myonuclei than *Dysf* muscle. (**F**) *DysfA6* had reduced muscle function, demonstrated by reduced normalized peak torque, compared with *Dysf* muscle. (**G**) *DysfA6* mice had reduced voluntary activity compared with *Dysf* mice, seen as reduced time spent in stereotypy behaviors and vertical movements. (**H**) Serum creatine kinase (CK), an indicator of muscle leak, was greater in *DysfA6* mice compared with *Dysf* mice (*n* = 4–7 mice per genotype). *P < 0.05; **P < 0.01; ***P < 0.005; ****P < 0.001 by 2-tailed Student’s *t* test for comparison between 2 groups (**B**–**F**) and 1-way ANOVA for comparisons across 3 groups (**G** and **H**) followed by Tukey’s multiple comparisons test. Scale bars: 100 μm.

Dysferlin and ANXA6 participate in plasma membrane repair and resealing after injury, while dystrophin and sarcoglycans maintain membrane stability (Figure 1A). To understand the role of ANXA6 in muscle, we characterized muscle histopathology in *Anxa6^–/–^* mice (39), in which exon 3 of *Anxa6* was disrupted. These mice, referred to as *A6^–/–^* mice, demonstrated few pathological findings (Supplemental Figure 1; supplemental material available online with this article; https://doi.org/10.1172/jci.insight.205137DS1). ANXA1 was mildly increased in *A6^–/–^* muscle (Supplemental Figure 1B). To determine whether ANXA6 contributes to membrane resealing in coordination with dysferlin, we crossed *A6^–/–^* mice with *Dysf* mice that lack dysferlin implicated in membrane repair, producing *DysfA6* mice. Compared with *Dysf* muscle, *DysfA6* muscle exhibited a pronounced increase in mononuclear infiltration, including PDGFRα^+^ fibroblasts, interstitial fibrosis, and fiber size variability (Figure 1B). *DysfA6* muscle had an increase in smaller myofibers and variability in myofiber size, and a 3-fold increase in myofibers with centralized nuclei compared with *Dysf* muscle (Figure 1, C–E, and Supplemental Figure 2). *DysfA6* mice also had reduced body weight–normalized peak torque and voluntary movement, measured by stereotypical and vertical movements (Figure 1, F and G). Serum creatine kinase (CK) levels, a clinical marker indicative of muscle injury and sarcolemmal leak, was elevated in *DysfA6* mice compared with wild-type (WT) and *Dysf* mice (Figure 1H).

**Figure 2:**
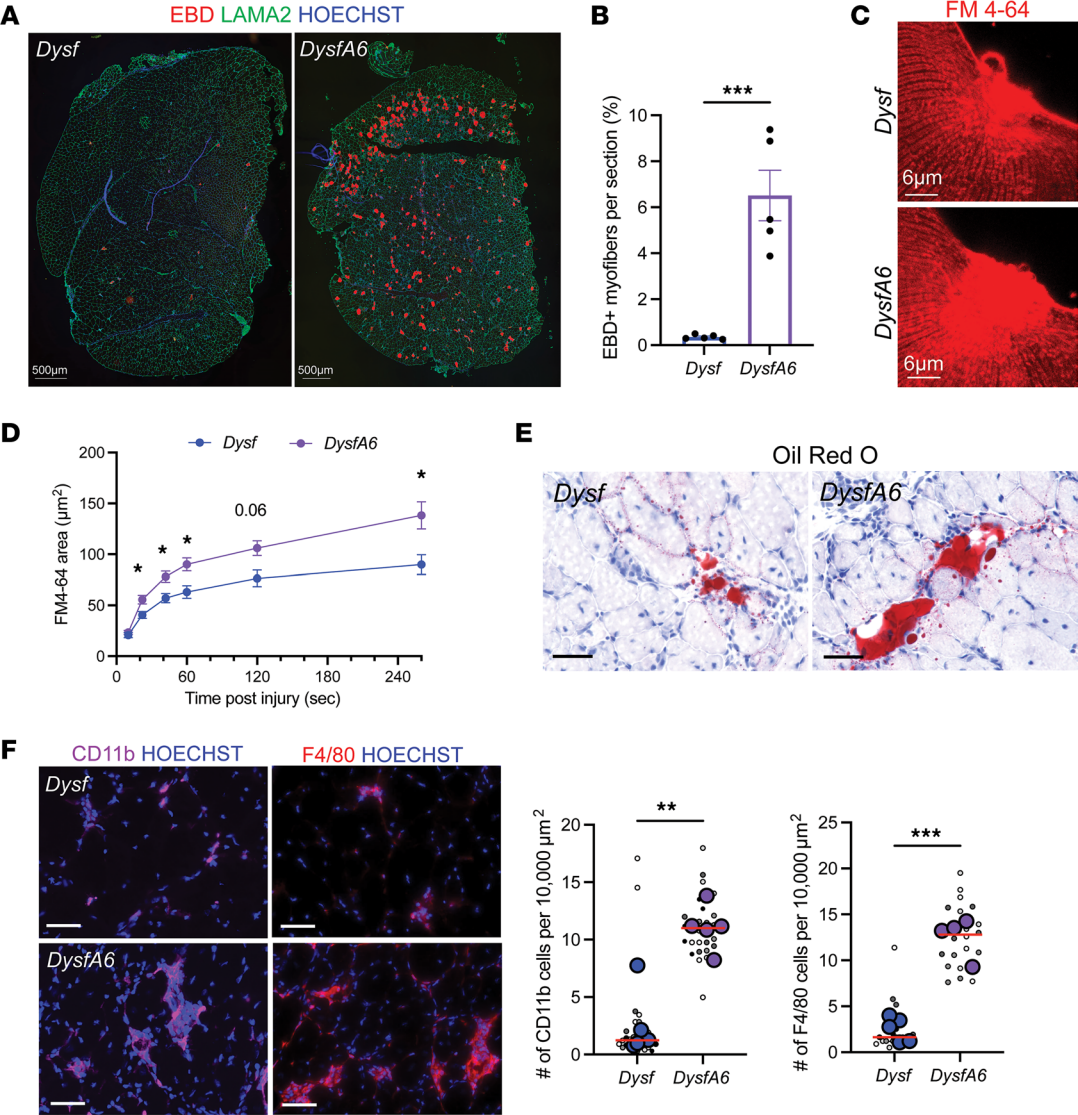
Muscle lacking both dysferlin and ANXA6 (*DysfA6* muscle) has more sarcolemmal leak, delayed sarcolemmal repair, and more macrophage infiltration compared with *Dysf* muscle. (**A**) Mice were injected with Evans blue dye (EBD), and red EBD^+^ myofibers were visualized, demonstrating more EBD^+^ fibers in *DysfA6* quadriceps muscles compared with *Dysf* muscle. LAMA2 (green) outlines the myofibers. (**B**) Quantification of EBD^+^ myofibers in *Dysf* and *DysfA6* muscle (*n* = 5 mice per genotype, 2 sections per mouse). (**C**) Myofibers were isolated from flexor digitorum brevis muscle and injured using a laser in the presence of FM 4-64 dye (red) to mark the injury area. Representative image of laser-injured *Dysf* and *DysfA6* myofibers with injury area marked in red, showing increased FM 4-64 area in *DysfA6* myofibers compared with *Dysf* myofibers. (**D**) Kinetics analysis showing all pairwise comparison at each assessed time point demonstrating greater FM 4-64 area in *DysfA6* than *Dysf* myofibers (*n* = 20 total myofibers from *n* = 3 mice, 2 muscles each, total 6 muscles), consistent with delayed and inefficient myofiber repair. (**E**) *DysfA6* muscle had more neutral lipid deposits (Oil Red O) than *Dysf* muscle. (**F**) *DysfA6* muscle had more leukocyte and macrophage infiltration (CD11b and F4/80, respectively) compared with *Dysf* (counts per 10,000 μm^2^). Small dots depict counts from each field of view (*n* = 5 mice per genotype, 5 images across 2 sections per mouse); large dots depict average per mouse. **P < 0.01, ***P < 0.005 by 2-tailed Student’s *t* test for comparisons between averages per mouse. Scale bars: 500 μm (**A**), 6 μm (**C**), and 100 μm (**E** and **F**).

To investigate the effect of delayed sarcolemmal resealing in *DysfA6* muscle, we used Evans blue dye (EBD) and visualized the spatial distribution of EBD^+^ fibers (40). *DysfA6* muscle had 19-fold more EBD^+^ myofibers than *Dysf* muscle (Figure 2, A and B). We used a laser to disrupt the sarcolemma of isolated *Dysf* and *DysfA6* myofibers in the presence of FM 4-64 dye, which marks membrane lesions. Over 260 seconds after injury, *DysfA6* myofibers consistently displayed increased FM 4-64 in all pairwise comparisons compared with *Dysf* myofibers, consistent with slower membrane resealing in *DysfA6* myofibers (Figure 2, C and D). Additionally, neutral lipids were increased in *DysfA6* compared with *Dysf* muscle, as seen by Oil Red O staining, suggesting that the loss of ANXA6 further exacerbates altered lipid handling in *Dysf* muscle (41, 42) (Figure 2E). *DysfA6* muscle had increased immune infiltration marked by CD11b, a marker of leukocytes, and F4/80, a marker of macrophages, relative to *Dysf* muscle (Figure 2F). Together, these data demonstrate a synthetic effect of eliminating 2 distinct membrane repair proteins, ANXA6 and dysferlin, slowing membrane resealing, promoting membrane leak, and eliciting heightened inflammatory cell infiltration.

### Fibroblasts/fibro-adipogenic precursors and macrophages/myeloid cells are the predominant mononuclear cell type in Dysf and DysfA6 muscle.

**Figure 3:**
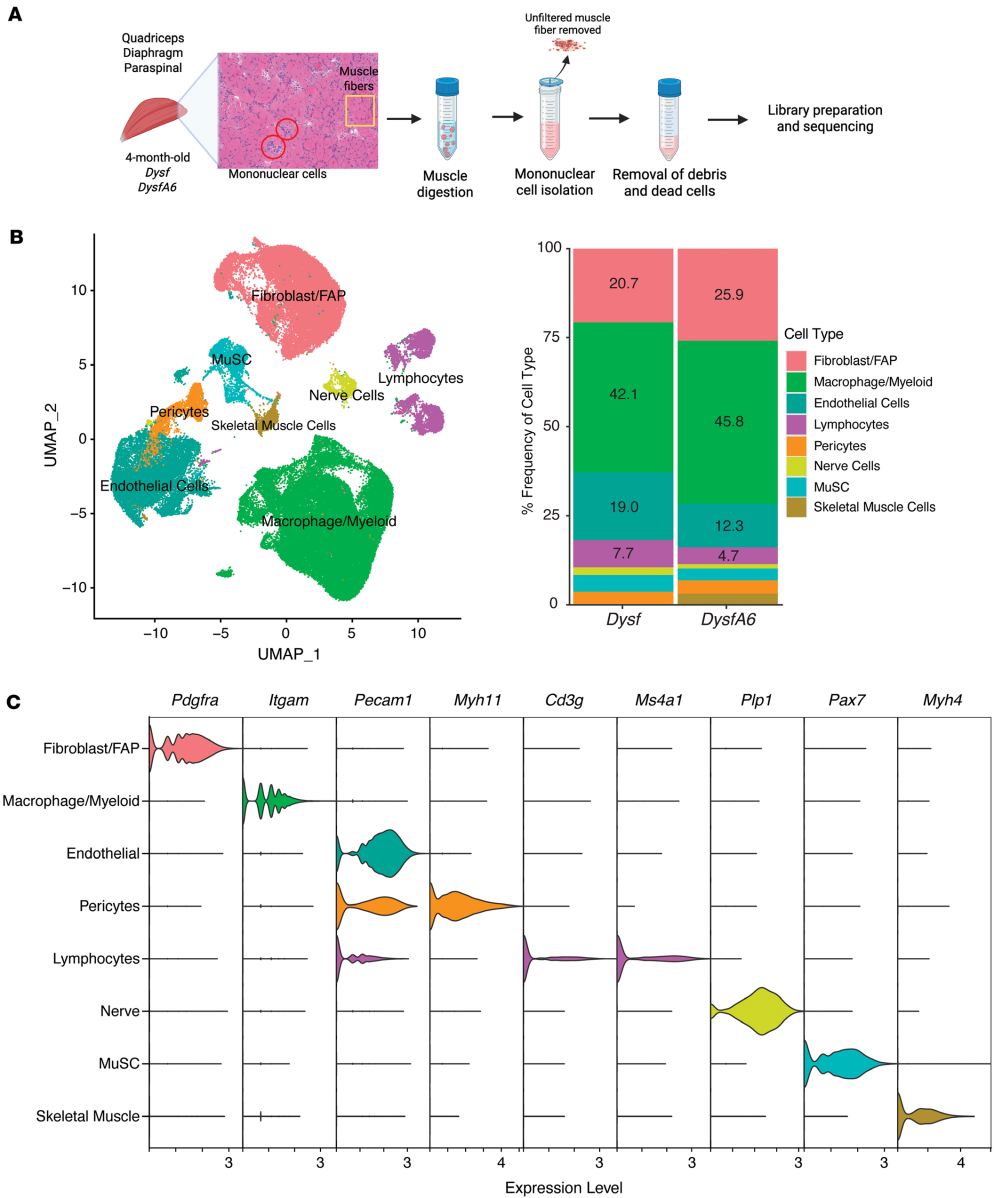
Fibroblasts/FAPs and macrophages/myeloid cells dominate the mononuclear cell populations in *Dysf* and *DysfA6* muscle. (**A**) *Dysf* and *DysfA6* muscles were homogenized, combined, and large myofibers were removed using size exclusion filtration. The unselected, mononuclear cell fraction was then analyzed by scRNA-seq (~20,000 cells per mouse, 2 mice of each genotype, total of ~82,000 cells). (**B**) UMAP analysis identified 8 major cell types comprising the mononuclear cell fraction with macrophages/myeloid cells and fibroblasts/FAPs contributing to greater than 60% of the mononuclear cells. (**C**) Violin plots depict canonical gene expression markers for the cell populations in **B**. MuSC, muscle satellite cells.

This heightened inflammatory infiltration both reflects, and likely, contributes to the more intense pathology in *DysfA6* muscle. To characterize this response, the mononuclear cell fraction of muscle was isolated and analyzed by single-cell RNA-seq (scRNA-seq). Multiple muscles were combined, including the quadriceps, diaphragm, and paraspinal muscles from 4-month-old *Dysf* and *DysfA6* mice, and muscles were digested and filtered to remove large muscle fibers, leaving mononuclear cells with greater than 80% viability (*n* = 2 per genotype, 4 mice total). Samples from each mouse were separately processed on the droplet-based platform, sequenced, and then data were combined; this strategy mitigates biological variability across muscle groups and animals (Figure 3A). Uniform Manifold Approximation and Projection (UMAP) and unbiased clustering analysis of 82,370 mononuclear cells (*Dysf* = 41,655, *DysfA6* = 40,715) identified 8 major cell types based on known marker gene expression. In this analysis, platelets were removed, and tenocytes were combined with fibroblasts. We confirmed a consistent distribution of different cell types across the 2 mice for each genotype. In both *Dysf* and *DysfA6* muscle, the macrophage/myeloid populations constituted the largest subtype, followed by fibroblast/fibro-adipogenic precursor (FAP), and together these groups contributed to the more than 60% of the mononuclear cells isolated from muscle (Figure 3, B and C).

### Mertk and Trem2 expression is enriched in Dysf and DysfA6 muscle myeloid cells compared with mdx muscle.

**Figure 4:**
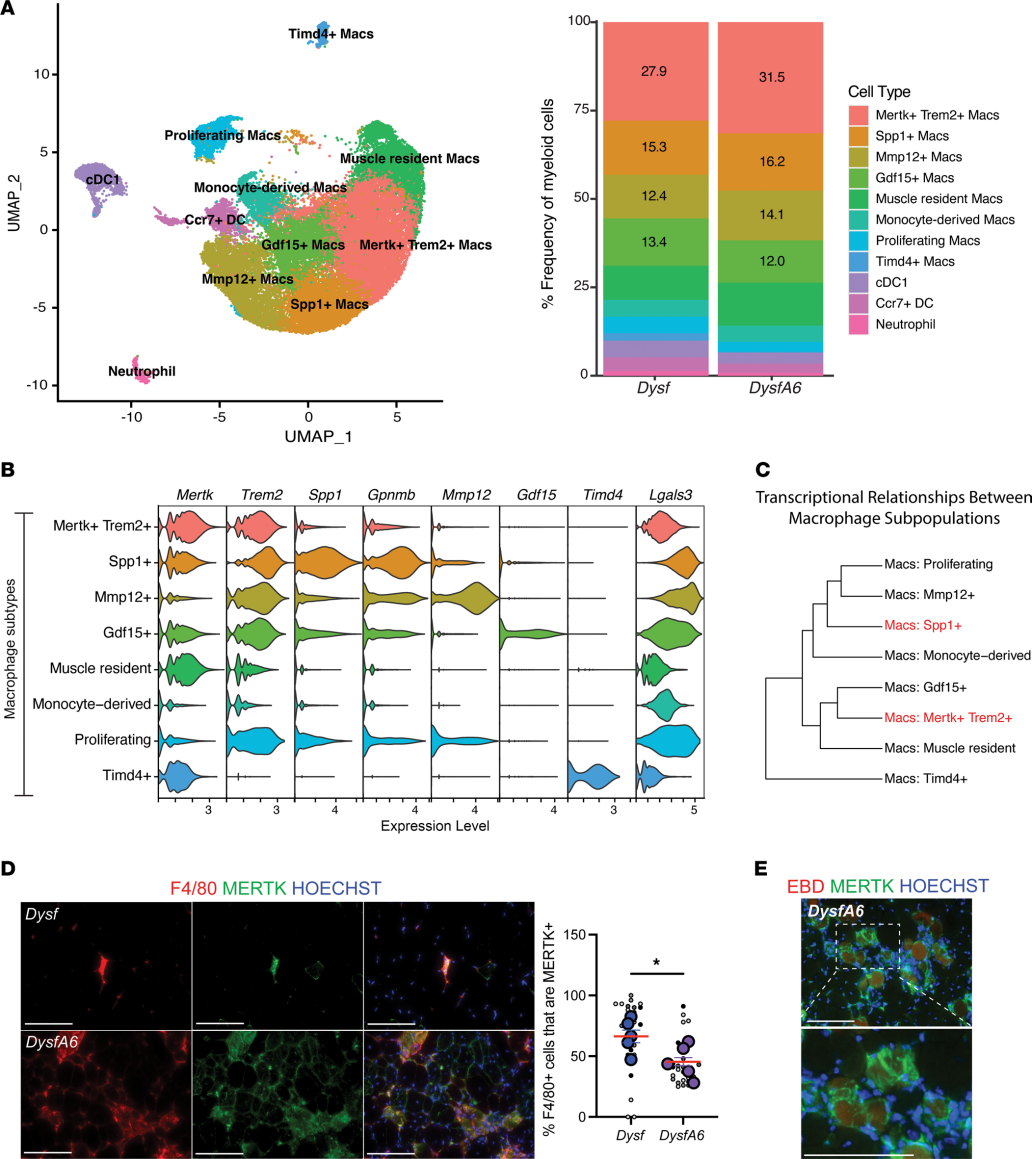
*Mertk^+^Trem2^+^* macrophages are the largest subtype of macrophages. (**A**) *Adgre1*^+^ (F4/80) myeloid cells from the *Dysf* and *DysfA6* mononuclear cell fraction were analyzed by UMAP to identify macrophages, neutrophils, and dendritic cells. *Mertk^+^Trem2^+^* macrophages constitute the largest subtype. (**B**) Expression of select genes across macrophage subtypes, highlighting the relatively low *Spp1* expression in *Mertk^+^Trem2^+^* macrophages. (**C**) The dendrogram based on gene expression profiles identifies relationships among macrophage subtypes showing that *Mertk*^+^*Trem2*^+^ macrophages are distinct from *Spp1*^+^ macrophages. (**D**) MERTK protein signal (green) was more abundant in *DysfA6* compared with *Dysf* muscle, and approximately 50%–60% of macrophages expressed MERTK in *Dysf* and *DysfA6* muscle (*n* = 5 mice per genotype, 4–5 images across 2 sections per mouse). (**E**) MERTK (green) signal clustered around EBD^+^ (red) myofibers in *DysfA6* muscle. *P < 0.05 by 2-tailed Student’s *t* test for comparisons between averages per mouse. Scale bars: 100 μm.

We analyzed the macrophage/myeloid cell population from *DysfA6* and *Dysf* muscle. Based on known marker gene expression, we identified 11 different myeloid cell populations, ranging from subtypes of macrophages to dendritic cells and neutrophils. In both models, the *Mertk*^+^*Trem2*^+^ macrophage population was the largest myeloid cell group, followed by *Spp1*^+^ macrophages (Figure 4A and Supplemental Figure 3, A and B). TREM2 is a lipid-binding receptor, and MERTK works together with TREM2 to facilitate phagocytosis (43, 44). MERTK, a member of the TAM receptor family, mediates the recognition and engulfment of injured cells through interaction with the externalized PS, an “eat-me” signal on the cell membrane (21, 27). Notably, the macrophage subpopulations did not group by the canonical M1 and M2 classification and instead displayed a broader diversity of subtypes (Supplemental Figure 3C).

Duchenne muscular dystrophy (DMD), a distinct genetic disorder, is caused disruption of the dystrophin gene (4), producing a fragile sarcolemma distinct from defective repair in dysferlinopathy (6). In *mdx* mice, a DMD model, *Spp1^+^Lgals3^+^* macrophages were identified as the dominant pathological macrophage population (33, 38). While this subgroup was present, *Mertk^+^Trem2^+^* macrophages constituted the largest macrophage population in *Dysf* and *DysfA6* muscle. *Mmp12^+^Gdf15^+^*, muscle-resident, monocyte-derived, proliferating macrophages were in common to *Dysf*, *DysfA6*, and *mdx* (33, 36). *Timd4^+^* macrophages, which were identified in acutely injured muscle (45), were also present in *Dysf* and *DysfA6* muscle (Figure 4B). Hierarchical dendrogram clustering delineates the transcriptional relationships between macrophage subpopulations and confirmed that *Mertk^+^Trem2^+^* is divergent from *Spp1^+^* macrophages, consistent with being distinct subtypes (Figure 4C). Immunofluorescence microscopy (IFM) imaging of MERTK protein in muscle revealed that MERTK was expressed in approximately 50%–60% of *Dysf* and *DysfA6* F4/80*^+^* macrophages (Figure 4D). Although *Dysf* exhibited a higher proportion of MERTK*^+^* macrophages than *DysfA6*, this is likely attributable to the significantly lower overall macrophage abundance in *Dysf* muscle (Figure 2F). MERTK^+^ cells clustered around EBD^+^ myofibers in *DysfA6* muscle (Figure 4E), consistent with leaking fibers as macrophage attractants. Together, these data document that excess membrane leak driven by impaired membrane repair elicits distinct major macrophage populations compared with *mdx* muscle, suggesting the mode of muscle injury produces a differential macrophage response.

### mdx muscles have minimal MERTK expression around damaged fibers.

**Figure 5:**
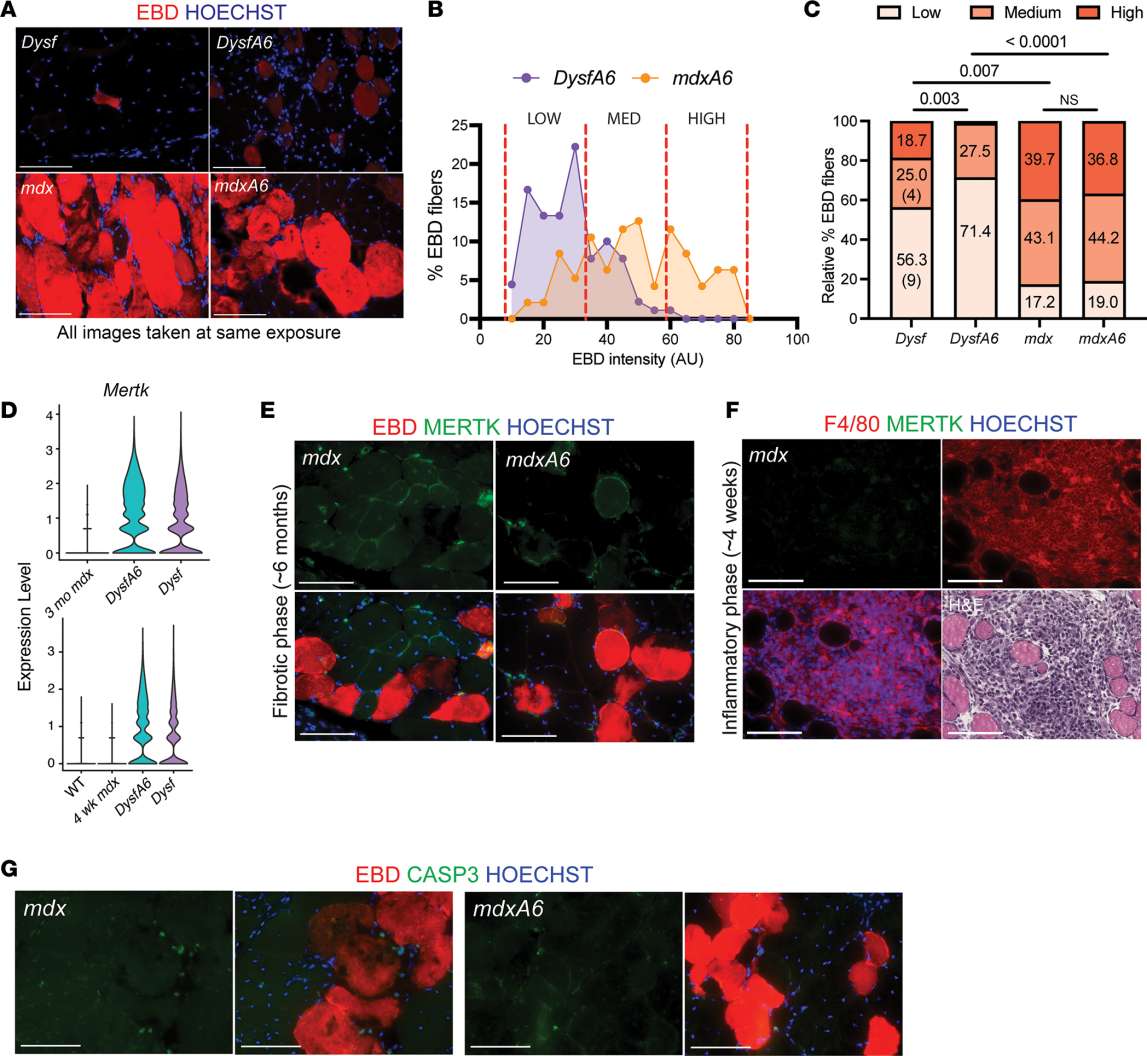
EBD uptake patterns differ between *mdx* and *Dysf* myofibers. (**A**) *Dysf*, *DysfA6*, *mdx*, and *mdxA6* mouse muscles analyzed after EBD injections. *mdx* and *mdxA6* muscle had brighter, more intense clusters of EBD^+^ fibers compared with the diffuse, light EBD uptake pattern seen in *Dysf* and *DysfA6* muscle. (**B**) The distribution of EBD intensity across muscle sections differed, with *DysfA6* muscle displaying more fibers with low EBD intensity compared with the high EBD intensity seen in *mdxA6*. (**C**) The relative proportion of low, medium, and high EBD intensity myofibers is shown for the 4 genotypes, *Dysf*, *DysfA6*, *mdx*, and *mdxA6* (*n* = 3 mice per genotype, 4–5 images from 2 sections per mouse). *P* values derived from the χ^2^ test. (**D**) *mdx* muscle also had little *Mertk* expression compared with *Dysf* and *DysfA6*. The plots show comparably analyzed *mdx* scRNA-seq (33, 38), compared to *Dysf* and *DysfA6*. (**E**) MERTK (green) was not detected around EBD^+^ (red) fibers in *mdx* and *mdxA6* muscle (6-month-old animals). (**F**) Area of high macrophage infiltration (red) in 4-week-old *mdx* muscles also did not show MERTK expression (green). (**G**) CASP3 staining was not detected in EBD^+^ (red) myofibers from *mdx* and *mdxA6* muscle. Scale bars: 100 μm.

We also generated *mdxA6* mice that lacked both dystrophin and ANXA6. Unlike *Dysf*, loss of ANXA6 in *mdx* mice did not exacerbate disease pathology, as inflammation and fibrosis were similar to those in *mdx* muscle (Supplemental Figure 4). EBD uptake into myofibers occurs in dystrophin-deficient muscle, related to the fragile sarcolemma (46). We compared myofiber leak, reflected by EBD intensity, across the 4 models since EBD has been shown to distinguish among types of cellular injury (47). Mice from all 4 genotypes were injected with EBD, and muscles were harvested and imaged identically. *mdx* and *mdxA6* muscle had strongly EBD-positive fibers, often clustered into groups of very bright EBD signal. This pattern differed from EBD uptake in *Dysf* and *DysfA6* muscle, where many more individual EBD^+^ fibers with much lower EBD intensity were broadly distributed across the muscle (Figure 5, A–C). We interpreted the brightly stained EBD^+^ myofibers in *mdx* and *mdxA6* as reflecting a larger-scale membrane disruption from the *mdx*’s inherent sarcolemmal instability. In contrast, *Dysf* and *DysfA6* have a more distributed, lighter EBD pattern, reflecting smaller-scale injury with resealing defects and prolonged leak. While delayed repair may allow additional EBD uptake, the increased frequency of low-intensity EBD^+^ fibers in *DysfA6* relative to *Dysf* suggests that the sustained membrane leak is the predominant factor underlying this observation.

We queried scRNA-seq datasets of *mdx* skeletal muscle at different disease stages (33, 38). We found minimal *Mertk* expression in *mdx* macrophages at both 3 months, an age of established disease, and 4 weeks, an age of peak inflammation, compared with *Dysf* and *DysfA6* macrophages, which was confirmed by IFM imaging (Figure 5, D–F). IFM showed that EBD^+^ fibers in *mdx* and *mdxA6* muscle did not express cleaved CASP3, supporting previous findings that a caspase-independent, necroptotic process is responsible for myofiber death in *mdx* muscle (48) (Figure 5G). The slow-leak nature of myofiber injury in *Dysf* and *DysfA6* muscle instead is associated with MERTK^+^ macrophages, similar to what has been described in other acute-tissue-injury settings (28–30).

### Impaired sarcolemmal repair leads to increased cleaved CASP3 and elevated macrophage-mediated phagocytosis.

**Figure 6:**
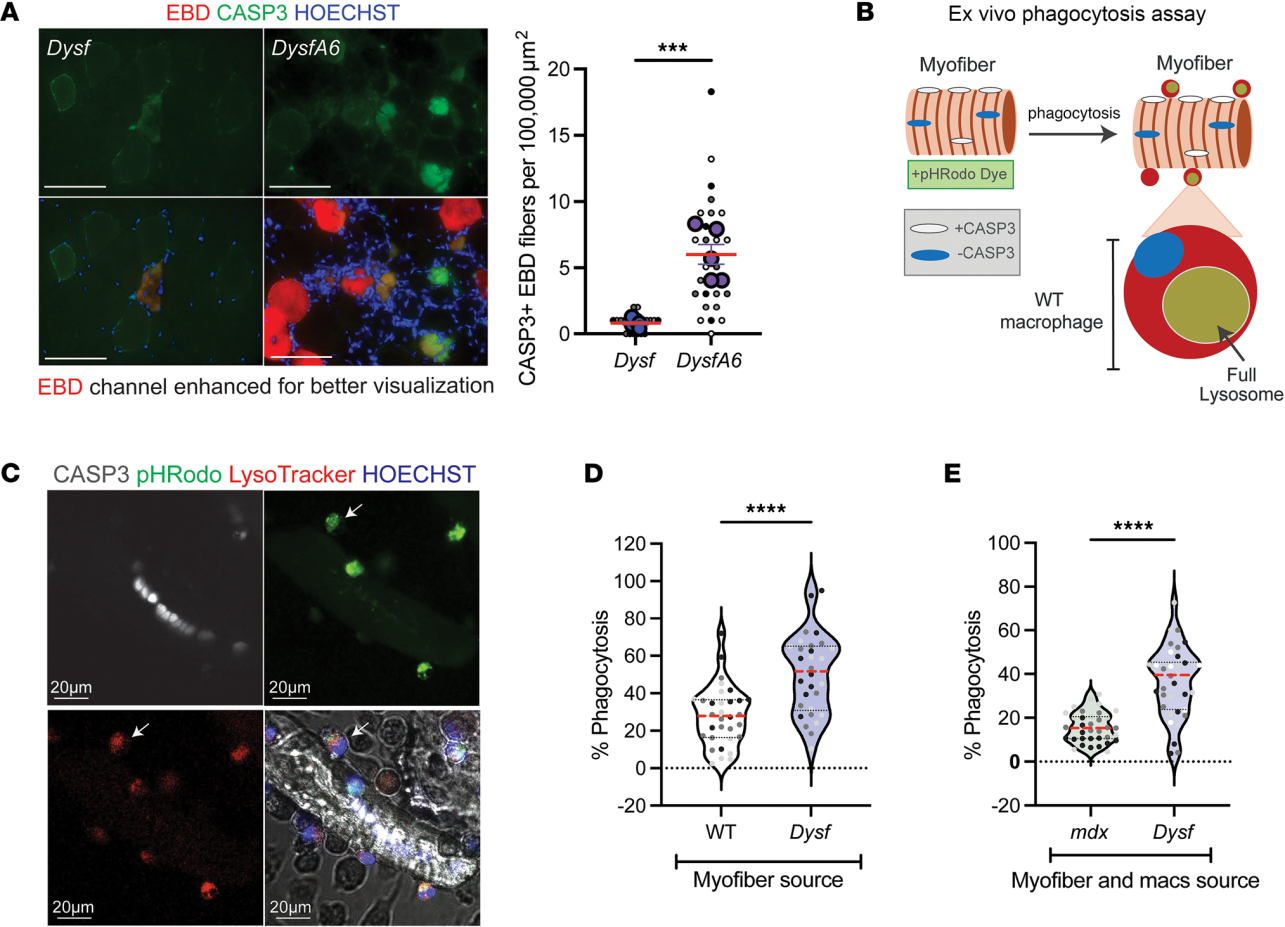
Enhanced CASP3 expression and macrophage phagocytosis of myofibers with impaired membrane resealing. (**A**) Representative IFM images of EBD-injected *Dysf* and *DysfA6* muscle stained for cleaved caspase-3 (CASP3) demonstrating a subset of EBD^+^ fibers are CASP3^+^. Statistical test depicts comparison between averages per mouse. (**B**) Myofibers were isolated from WT and *Dysf* mice and loaded with pHRodo dye. WT macrophages were isolated, differentiated, and then stained with LysoTracker (red). Myofibers were incubated with macrophages, and macrophages positive for pHRodo (green) had phagocytosed myofibers. Percentage macrophage phagocytosis was determined by the number of pHRodo^+^ macrophages divided by the total number of macrophages on or near CASP3^+^ myofibers. (**C**) Representative image of a CASP3^+^ myofiber and macrophages that were both LysoTracker^+^ and pHRodo^+^, indicative of phagocytosis (arrow). (**D**) *Dysf* myofibers triggered a greater fraction of phagocytic macrophages compared with WT myofibers. (**E**) *Dysf* myofibers triggered more phagocytosis compared with *mdx* myofibers. Ex vivo phagocytosis assay was conducted 3 different times. ***P < 0.005; ****P < 0.001 by 2-tailed Student’s *t* test. Scale bars: 100 μm (**A**) and 20 μm (**C**).

To further explore the intrinsic differences in the myofibers, we performed bulk RNA-seq on the myofibers isolated from *Dysf* and *DysfA6*; WT myofiber RNA-seq data were previously generated using identical methods (49) and enrichment of the myofibers was confirmed by high expression of sarcomeric genes (Supplemental Figure 5, A and B). Pathway analysis revealed significant enrichment of efferocytosis pathway genes, including *Casp3* and *Pros1*, in *Dysf* compared with WT, and in *DysfA6* compared with *Dysf* myofibers (Supplemental Figure 5C). Activated CASP3 contributes to the externalization of PS (50), while *Pros1* encodes Protein S (PROS1), a secreted protein that serves as a ligand for TAM receptors critical for recognition of target cells with externalized PS, in addition to growth arrest–specific protein 6 (GAS6) (51, 52). In *Dysf* and *DysfA6* muscle, CASP3 signal was seen in and near EBD^+^ myofibers, especially those myofibers with lighter EBD positivity (Figure 6A), with a similar pattern for PROS1 (Supplemental Figure 5, D and E), consistent with defective repair yielding myofiber signals that draw macrophages primed for phagocytosis. This contrasts with minimal expression of MERTK, CASP3, and PROS1 in or near clusters of EBD+ myofibers in *mdx* and *mdxA6* muscle (Figure 5, D–G, and Supplemental Figure 6A), even in young, hyper-inflamed 4-week-old *mdx* muscle (Supplemental Figure 6, B–D). In *mdx* muscle, we noted more PROS1^+^EBD^+^ myofibers in the smaller and lighter EBD^+^ clusters in both *mdx* and *mdxA6* muscle, rather than the large patches of brightly positive EBD where PROS1 was not detected. This pattern is consistent with the concept that lower EBD positivity reflects fibers with delayed repair and not necessarily necrosis. We observed a similar pattern with CASP3 expression (Supplemental Figure 7).

To directly test whether macrophages could phagocytose *Dysf* myofibers, we adapted an ex vivo phagocytosis assay using bone marrow–derived macrophages from WT mice that were cultured and loaded with LysoTracker. LysoTracker-labeled macrophages were incubated with myofibers isolated from the flexor digitorum brevis (FDB) muscle of either WT or *Dysf* mice. The trituration process typically results in minor injury sufficient to artificially activate CASP3. After isolation, fibers were loaded with pHRodo dye that emits signal when exposed to lysosomal low pH. To measure phagocytosis, we monitored macrophages positive for both LysoTracker and pHRodo and near or touching CASP3^+^ myofibers (Figure 6B). *Dysf* fibers had more macrophages with LysoTracker^+^/pHRodo^+^ signals compared with WT, demonstrating impaired membrane repair generates enhanced “eat-me” signals, which then attract macrophages to digest *Dysf* muscle (Figure 6, C and D). We performed this ex vivo phagocytosis assay on myofibers from *mdx* and *Dysf* muscles, using macrophages derived from the same genotype source. We observed reduced phagocytosis of *mdx* fibers and macrophages compared with *Dysf* fibers and macrophages (Figure 6E). Although CASP3 activation was not seen as a feature of *mdx* muscle on IFM (Figure 5G), the fiber isolation protocol in the phagocytosis assay did elicit activation of CASP3. Despite the CASP3 activation ex vivo, phagocytosis of *mdx* myofibers by macrophages remained low (Figure 6E). While contribution from the disease-specific macrophages cannot be fully excluded, phagocytosis of *Dysf* myofibers was comparable between WT and *Dysf* macrophages, implying dysferlin-deficient myofibers promote phagocytosis (Figure 6, D and E). Taken together, these results show that impaired sarcolemmal repair and excessive membrane leak caused by dysferlin deficiency in myofibers contribute to the increased presence of phagocytic macrophages and promote their phagocytic function compared with the more necrotic *mdx* myofibers.

### Increased ANXA1 and ANXA2 deposited into DysfA6 ECM attracts macrophages and affects macrophage-FAP crosstalk.

**Figure 7:**
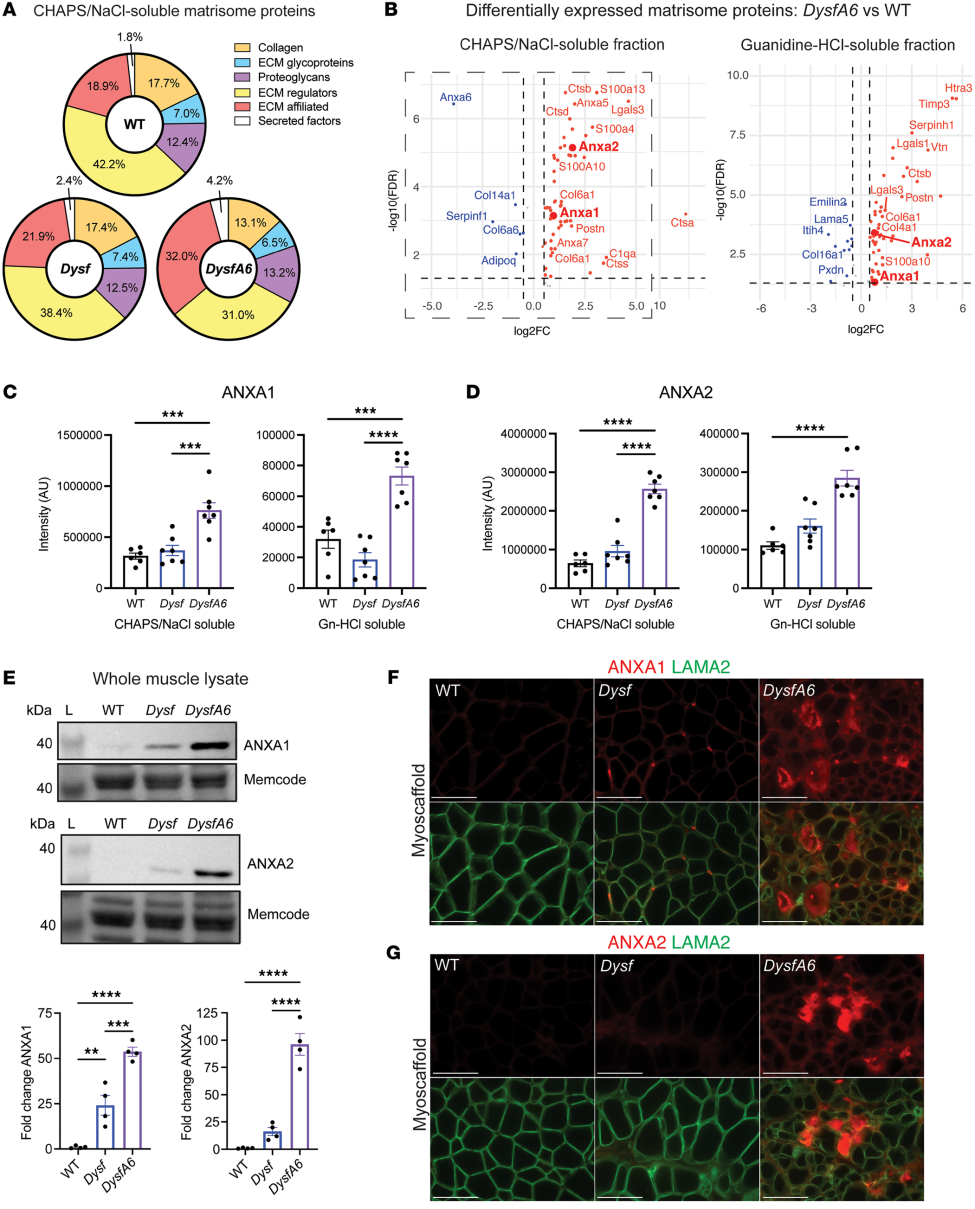
Increased ANXA1 and ANXA2 in *DysfA6* muscle and in decellularized myoscaffolds from *Dysf* muscle. (**A**) A comparison of proteomic profiles from the CHAPS/NaCl-soluble cellular fraction of WT, *Dysf*, and *DysfA6* muscle shows expansion of ECM-associated proteins (*n* = 6–7 mice per genotype). (**B**) Differentially expressed proteins between *DysfA6* and WT in the CHAPS/NaCl-soluble cellular and guanidine-HCl–soluble (Gnd-HCl-soluble) ECM fractions of the muscle. (**C** and **D**) ANXA1 and ANXA2 were increased in both the CHAPS/NaCl- and Gnd-HCl–soluble fractions of *DysfA6* compared with *Dysf* and WT (*n* = 6–7 mice per genotype). (**E**) Immunoblot of whole muscle lysates for ANXA1 and ANXA2 confirmed greater expression in the presence of defective repair. (**F** and **G**) Muscle sections were decellularized to generate myoscaffolds and visualize ECM-associated proteins. Increased ANXA1 and ANXA2 deposition was seen in decellularized myoscaffolds of *DysfA6* compared with WT and *Dysf* by IFM. **P < 0.01; ***P < 0.005; ****P < 0.001 by 1-way ANOVA followed by Tukey’s multiple comparisons test. Scale bars: 100 μm.

ANXA1 and ANXA2 support the ANXA6-rich repair cap (11, 12), and *Dysf* muscle ECM is enriched with ANXA2 and ANXA6 (53). To further understand how prolonged membrane leak alters the muscle ECM composition, we evaluated the ECM proteome (matrisome) of WT, *Dysf*, and *DysfA6* muscles using a 3-step ECM-optimized extraction method to characterize both cellular and ECM tissue fractions (54) (Figure 7A). Differentially expressed matrisome proteins between WT and *DysfA6* in the CHAPS/NaCl-soluble (cellular) and guanidine-HCl–soluble ECM fractions included ANXA1 and ANXA2 (Figure 7, B–D). Immunoblot analysis of whole muscle lysates confirmed that both ANXA1 and ANXA2 were increased in *DysfA6* muscle compared with WT and *Dysf* (Figure 7E). We decellularized muscle sections to generate myoscaffolds and confirmed the increased deposition of ANXA1 and ANXA2 in *DysfA6* ECM via IFM (Figure 7, F and G). Thus, the prolonged sarcolemmal leakage caused by defective membrane repair in *DysfA6* promotes excessive deposition of ANXA1 and ANXA2 in the muscle ECM.

**Figure 8:**
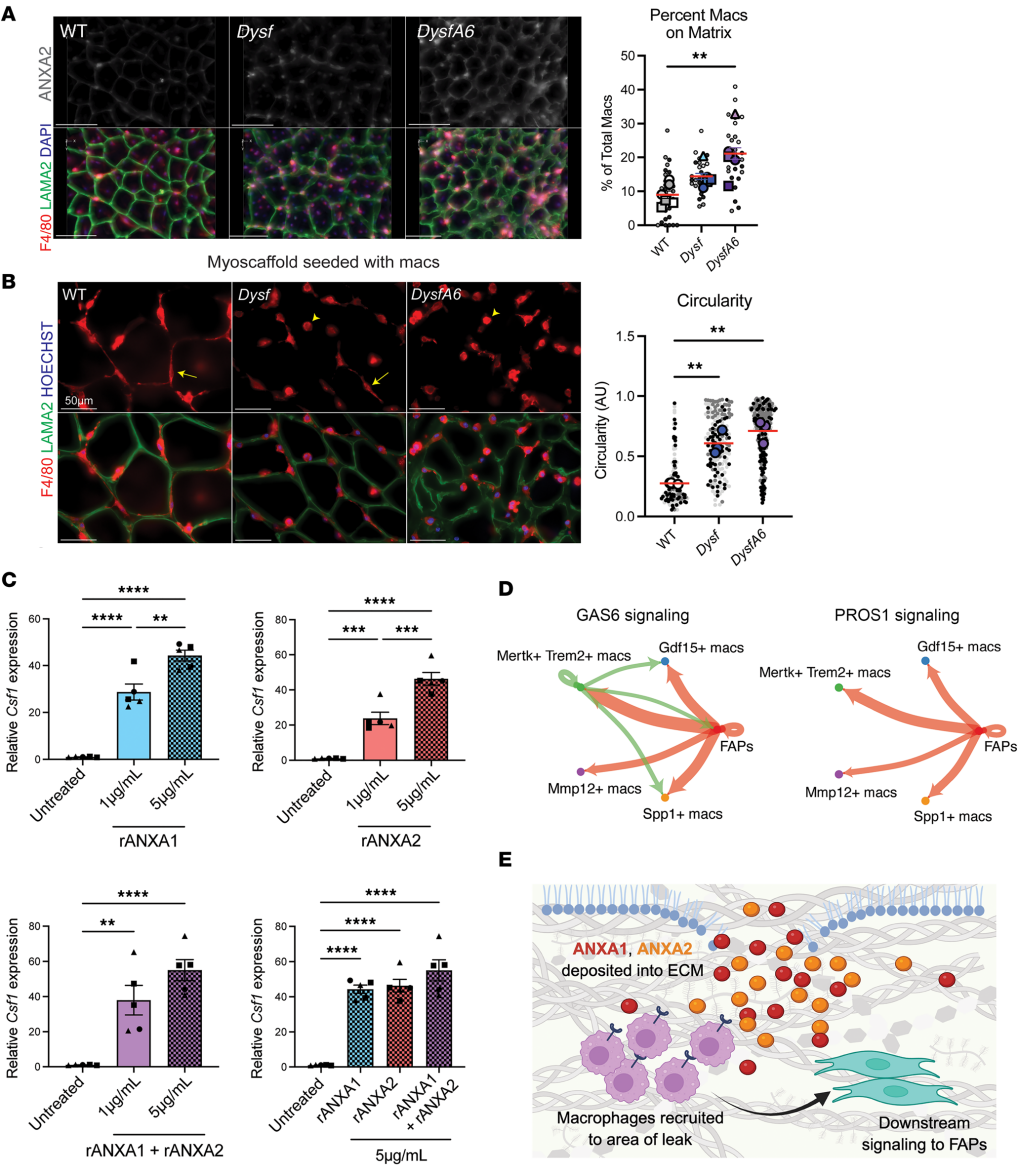
Elevated ANXA1 and ANXA2 expression in *DysfA6* ECM promotes expression of *Csf1* in macrophages. (**A**) Muscle cryosections (25 μm) were decellularized, incubated with WT macrophages, and imaged after 24 hours. *Z*-stack images were acquired and the percentage of macrophages localized on the ECM relative to the total macrophages on the ECM and glass was calculated. More macrophages (F4/80, red) accumulated on *Dysf* and *DysfA6* myoscaffolds compared with WT (*n* = 3 mice per genotype denoted by color, 2 myoscaffolds per mouse). Macrophages were derived from 3 WT mice, denoted by symbol shapes. (**B**) WT macrophages exposed to *Dysf* and *DysfA6* myoscaffolds adopted a distinct morphology compared with WT myoscaffolds, with greater increased circularity (arrowhead) induced by *Dysf* and *DysfA6* (*n* = 3 mice per genotype, 2 myoscaffolds per animal, and 3 images per myoscaffold). Full arrows indicate macrophages with more elongated morphology. (**C**) Recombinant ANX1 (rANXA1), rANXA2, or combined treatment of macrophages elicited *Csf1* expression (macrophages isolated from 3 mice, denoted by symbols, with technical replicates). (**D**) *DysfA6* receptor-ligand gene expression analysis with CellChat revealed that *Mertk^+^Trem2^+^* macrophages signal to FAPs via GAS6, while FAPs signal to macrophages via PROS1 and GAS6. (**E**) Prolonged sarcolemmal leak due to impaired membrane repair increased ANXA1 and ANXA2 ECM deposition, recruiting macrophages to the area of leak and promoting macrophage-FAP crosstalk. All the experiments were conducted 3 independent times. **P < 0.01; ***P < 0.005; ****P < 0.001 by 1-way ANOVA followed by Tukey’s multiple comparisons test. Scale bars: 100 μm (**A**) and 50 μm (**B**).

To investigate how altered ECM composition of *DysfA6* muscle influences macrophage behavior, WT macrophages were placed onto decellularized 25-μm-thick myoscaffolds for 24 hours, followed by *Z*-stack imaging. More macrophages were attracted to *Dysf* and *DysfA6* myoscaffolds compared with WT, with *DysfA6* myoscaffolds drawing more macrophages than *Dysf* (Figure 8A). Macrophages undergo a morphological change when becoming proinflammatory, adopting a “fried-egg” shape (55–57). When placed on *Dysf* and *DysfA6* myoscaffolds, macrophages became more circular compared with the elongated shape on WT myoscaffolds (Figure 8B). These data demonstrate that defective sarcolemmal repair alters the ECM composition, which is sufficient to attract macrophages and promote an altered profile.

We directly exposed macrophages to recombinant ANXA1 and ANXA2, finding dose-dependent effects on *Csf1* expression, and a trending additive effect of ANXA1 and ANXA2 (Figure 8C). *Csf1* encodes macrophage colony–stimulating factor (M-CSF), which is a chemoattractant factor that promotes survival and proliferation (58, 59). To further understand the signaling crosstalk between macrophages and FAPs, another prominent cell type involved in *Dysf* muscle pathology (60, 61), we analyzed gene expression for receptor-ligand pairs using CellChat cell-cell communication analysis, focusing on FAPs and *Mertk^+^Trem2^+^* macrophages that comprise 70% of the macrophages in *DysfA6* muscle. This interaction is projected to occur via genes implicated in GAS6 signaling, a pathway associated with TAM receptor–expressing macrophages and implicated in adipogenesis (62–66). FAPs signaled to macrophages via PROS1 and GAS6 pathways (Figure 8D). ANXA2 accumulation around myofibers is known to promote FAP differentiation to adipocyte-like lineages, and loss of ANXA2 is protective in dysferlinopathy (15, 60). Taken together, these data identify that defective membrane repair results in excess ANXA1 and ANXA2 in the ECM, which serves to recruit macrophages. Once recruited, macrophages differentiate and signal to FAPs via the GAS6 pathway, which in conjunction with excess ANXA2, could promote intramuscular fat deposition (Figure 8E).

## Discussion

### Disrupting the plasma membrane repair complex intensifies dystrophic pathology.

Global loss of ANXA2 impairs muscle membrane repair and muscle function (15), while global deficiency of ANXA1 showed minimal effect (67). Here we found global loss of ANXA6 minimally disrupts muscle pathology, yet in context with dysferlin deficiency, this promoted profound muscular dystrophy with excessive fibrosis and marked mononuclear cell infiltration. ANXA6 has been implicated in membrane repair within the nervous system, which was not examined in this study but may contribute to the changes in stereotypy function observed in *DysfA6* mice (10, 68–70).

Loss of ANXA6*,* in combination with dysferlin, leads to excessive ANXA2 protein in the ECM, supporting a pathological role for excessive ANXA2 in the ECM, consistent with the protective effect seen in *Dysf* muscle lacking ANXA2 (15). ANXA2 promotes adipogenesis in dystrophic muscle (60), consistent with the increased Oil Red O in *DysfA6* muscle. ANXA1 facilitates regeneration by resolving inflammation and promoting myogenic fusion (67, 71), but the excess ANXA1 in *DysfA6* seems unable to compensate for the excess ANXA2. ANXA6, ANXA1, and ANXA2 aggregate together in the repair cap (11, 12), and it is possible that annexins similarly aggregate in the ECM. ANXA6 can form exosomes during membrane repair (72, 73), which may be disrupted in *DysfA6* muscle.

### Dysferlin deficiency in muscle triggers phagocytosis.

Muscle expression of dysferlin is considered to be a primary driver of dysferlinopathy disease progression, as myofiber damage precedes macrophage infiltration and muscle-specific restoration of dysferlin expression was sufficient to reduce inflammation (18, 74). Adoptive transfer studies of dysferlin-deficient and WT bone marrow cells into *Dysf* mice demonstrated that the dysferlin-null environment, not the loss of dysferlin in the macrophages, contributed to macrophage recruitment and proliferation (75, 76). In our ex vivo phagocytosis assay, we observed that the *Dysf* myofiber enhanced macrophage phagocytosis, indicating altered myofiber signaling that affects macrophage function. Dysferlin is also involved in maintaining calcium homeostasis (77, 78), lipid handling (42, 79), vesicle fusion, and cytokine release (80–83), which can alter plasma membrane composition. These effects may also contribute to the observed increase in phagocytosis.

There is also evidence that macrophages themselves have abnormal features in dysferlinopathy. Patient-derived *DYSF* macrophages have increased motility (84). Macrophages of the A/J mouse, an inbred strain with spontaneous dysferlin deficiency, also have enhanced phagocytic capacity, although in the study by Nagaraju et al., the A/J mice lack a strain-matched control (85). We observed that phagocytosis of *Dysf* myofibers by WT and *Dysf* macrophages was comparable ex vivo, suggesting that the dysferlin deficiency has minimal effect on macrophage phagocytic capacity.

### Prolonged myofiber leak in dysferlinopathy influences macrophage heterogeneity.

We used differential EBD uptake into muscle to identify distinct myofiber leak profiles across the 2 models. Unlike the necrotic *mdx*, the leaky *Dysf* and *DysfA6* myofibers also displayed expression of activated CASP3 in situ, correlating with elevated MERTK expression surrounding the leaky myofibers and enhanced macrophage phagocytosis. Similarly, ex vivo, there was more phagocytosis of Dysf myofibers than mdx myofibers. This finding suggests that the leaky myofibers prime the macrophages to be more phagocytic. Classically, CASP3 activation occurs in apoptotic cells that lead to externalization of PS, marking the cell for efferocytosis (21). However, in skeletal muscle PS exposure by CASP3 activation also occurs in calcium overload and myogenic differentiation, processes commonly associated with myofiber injury, suggesting that the sustained membrane leak alone can trigger CASP3 activation (86–88). Furthermore, the annexin-rich repair cap has been shown to be PS-rich (73). Therefore, the expression of these markers in dystrophic muscle may not necessarily reflect apoptosis, but instead mark chronically leaky myofibers that attract the macrophages for phagocytosis of the repair cap.

### Excess leak alters the ECM, contributing to pathology.

*Dysf* muscle ECM has higher ANXA6 deposition than *mdx* ECM, and ANXA6 itself promotes myoblast motility and differentiation (53). Here, exacerbated leak in *DysfA6* in the ECM compared with *Dysf* led to elevated ANXA1 and ANXA2 deposition, which correlated with enhanced macrophage recruitment and altered, proinflammatory-like morphology. Macrophage morphology is known to be affected by ECM proteases needed for migration and infiltration into the tissue (89–91), suggesting that the dysregulated *DysfA6* ECM directly alters macrophage responses.

Previous studies demonstrated that endogenous and exogenous ANXA1 and ANXA2 influence macrophage polarization and function (71, 92–94). Here we found that ANXA1 and ANXA2 promoted expression of *Csf1*, which encodes M-CSF, a known stimulant of macrophage proliferation and survival (58, 59). Analysis of receptor-ligand gene pairs supported macrophage signaling pathways to FAPs, a cell population that is expanded in dysferlinopathy that contributes to the pathological ECM remodeling (60, 61). Collectively, our findings demonstrate that the prolonged leak due to defective membrane repair not only alters the muscle microenvironment but also redefines the immune-ECM interface, offering mechanistic insight into how defective repair propagates chronic inflammation and fibrosis in muscular dystrophy.

## Methods

### Sex as a biological variable.

Unless otherwise indicated, for WT, *Dysf*, and *DysfA6* mice, both male and female animals were used, as dysferlinopathy affects both male and female patients. For *mdx*, *mdxA6* males were used, as DMD is an X-linked genetic disease that only affects male patients.

### Animals.

Mice were housed in specific pathogen–free facility in accordance with Northwestern University’s Institutional Animal Care and Use Committee regulations. All mice used in the study are in the C57BL/6J background. B6.A-*Dysf^prmd^*/GeneJ (BLAJ) (95); referred to as *Dysf* here), C57BL/10ScSn-*Dmd^mdx^*/J (*mdx*), and WT C57BL/6J mice were obtained from The Jackson Laboratory (strains 012767, 001801, and 000664). *A6^–/–^* mice were previously generated by inserting a neomycin cassette into exon 3, generating a null allele (39), and these mice were derived using B6;129P2-*Anxa6*^tm1Moss^/H sperm from EMMA (EM:09630) and re-derived at Northwestern University’s Transgenic and Targeted Mutagenesis Laboratory (TTML). Studies were conducted on 4- to 6-month-old mice.

### Genotyping.

Tail DNA was genotyped using the following primer set flanking *Anxa6* exon 3: primer F 5′-GGACCCGCTCTTGGGTAAC-3′ and primer R 5′-CAGGGTCCAGGTCAAAGGT-3′.

### Immunoblotting.

Tissues were lysed in whole tissue lysis buffer composed of 50 mM HEPES pH 7.5, 150 mM NaCl, 2 mM EDTA, 10 mM NaF, 10 mM Na-pyrophosphate, 10% glycerol, 1% Triton X-100, 1 mM phenyl-methylsulfonyl fluoride (PMSF), and 1× cOmplete Protease Inhibitor Cocktail (11697498001 CO-RO, Roche). Lysates were separated in 4%–15% Mini-PROTEAN TGX Precast Protein Gels (4561086, Bio-Rad Laboratories) and transferred to Immun-Blot PVDF Membranes (1620177, Bio-Rad Laboratories). Blocking and antibody incubations were done using StartingBlock T20 (TBS) Blocking Buffer (37543, Thermo Fisher Scientific). Secondary antibodies conjugated to horseradish peroxidase were used at a dilution of 1:2500. Antibodies are listed in Supplemental Table 1.

### IFM, cell count, and colocalization analysis.

Muscle sections (10 μm) were fixed with 4% paraformaldehyde (PFA) (15710, Electron Microscopy Sciences). Samples were blocked for 1 hour in 1% BSA with 10% fetal bovine serum and 0.1% Triton X-100. Primary antibodies were used at 1:100 dilution overnight and secondary antibodies were at 1:2500 with a 1-hour incubation at room temperature. Five representative ×40 images across 2 muscle sections were chosen and the number of nuclei within areas positive for the cell type marker was counted. To quantify the percentage of F4/80^+^ areas also positive for MERTK, IFM images were overlaid and loaded into a Keyence BZ-X800 analyzer, and analyzed under Hybrid Cell Count, Single Extraction option under the same condition.

### Cross-sectional area and internal nuclei analyses.

Muscle sections were stained with anti–laminin-2 (anti-LAMA2) to outline myofibers, and Hoechst stained quantified using Myotally (96).

### In vivo tetanic torque.

Peak plantar complex tetanic isometric torque was assessed using a Whole Mouse Test System (1300A, Aurora Scientific) equipped with a 1 N footplate force transducer (300C-LR, Aurora Scientific). Bipolar silver electrodes were inserted subcutaneously over the tibial nerve, and muscle contractions were evoked using a 0.1 ms pulse width and 0.3 second train duration. A force–frequency curve was constructed by applying a series of stimulation frequencies (10, 40, 80, 125, and 200 Hz), with 30 seconds of rest between contractions, from which peak tetanic torque was identified. Studies were conducted on approximately 10-month-old *Dysf* and *DysfA6* mice, with equally balanced male and female mice.

### Activity assessment.

Unanesthetized mouse activity was measured individually using an Omnitech Open Field activity cage (Omnitech Electronics) across 48 hours, and data were analyzed during the night cycle (7 pm to 7 am) on the second night of acquisition.

### EBD injection and intensity quantification.

EBD uptake into muscle was quantified as described previously (12, 14). Each EBD^+^ myofiber was traced and the mean intensity was measured using FIJI (NIH). When comparing *Dysf*, *DysfA6*, *mdx*, and *mdxA6*, EBD fibers were imaged at the same exposure. When comparing *Dysf* against *DysfA6*, the EBD intensity was enhanced for better visualization. Based on the lowest and the highest intensity, EBD intensity was divided into 3 levels, low, medium, and high. The number of EBD myofibers that fell within each intensity level was quantified and used for the χ^2^ test.

### FDB myofiber isolation and laser injury.

Fibers were dissected and laser damaged as described previously (11, 97). FM 4-64 dye (T-13320, Molecular Probes) was added to a final concentration of 2.5 mM prior to imaging. Fibers were wounded at room temperature using a Nikon A1R laser-scanning confocal microscope equipped with GaSP detectors through a 60× Apo lambda 1.4 NA objective driven by Nikon Elements AR software. Ablation was performed using a single pixel set as 120 nm (0.0144 mm^2^) with the 405 nm laser at 100% power for up to 5 seconds. Kinetic analysis was conducted by assessing the fluorescence area of the lesion at 10, 22, 42, 60, 120, and 260 seconds after the injury.

### Mononuclear cell isolation.

Quadriceps, abdominal, and paraspinal muscles were freshly harvested from 4-month-old female *Dysf* and *DysfA6* (*n* = 2 each, 4 mice total), washed in sterile-filtered Dulbecco’s PBS (DPBS) with calcium and magnesium (MT21030CV, Thermo Fisher Scientific), combined, and weighed. The tissues were minced in 6 mg/mL collagenase type II (LS004177, Worthington) solution in a 1:5 weight to volume ratio and enzymatically digested at 37°C for 1.5 hours with shaking. The digested tissues were then filtered through 70 μm nylon filters and washed with calcium- and magnesium-free DPBS (14190144, Thermo Fisher Scientific). Debris was removed from the samples using Debris Removal Solution (130-109-398, Miltenyi Biotec) following the manufacturer’s instructions. The subsequent cell pellet was resuspended in 1× TheraPEAK ACK lysing buffer (BP10-548E, Lonza) to remove the red blood cells, filtered through 40 μm nylon filter, and resuspended in 1% BSA/DPBS solution. The final cell suspension was diluted 1:10, mixed 1:1 with ViaStain AOPI Staining Solution (CS2-0106-5ML, Revvity), and counted on a Nexcelom Cellometer Auto 2000 to ensure viability greater than 70%.

### scRNA-seq and analysis.

Mononuclear cells were analyzed using a Nexcelom Cellometer Auto 2000 with AOPI fluorescent staining and cells (29,000) were loaded into the Chromium iX Controller (PN-1000328, 10X Genomics) on a Chromium GEM-X Single cell 3′ Chip Kit v4 (PN-1000215, 10X Genomics), and processed to generate single-cell gel beads in the emulsion (GEM). cDNAs and library were generated using the GEM-X Single cell 3′ Kit v4 (PN-1000691, 10X Genomics) and Dual Index Kit TT Set A (PN-1000215, 10X Genomics). Quality control for the constructed library was performed by Agilent Bioanalyzer High Sensitivity DNA kit (5067-4626, Agilent Technologies) and Qubit DNA HS assay kit for qualitative and quantitative analysis, respectively. Multiplexed libraries were pooled and sequenced on an Illumina NovaSeq X Plus sequencer with 100-cycle kits using the following read length: 28 bp Read1 for cell barcode and UMI, and 90 bp Read2 for transcript. The single-cell library preparation and sequencing were done at the Northwestern University NUseq facility core. Sequence reads were aligned to the mm10 mouse reference genome using the CellRanger Count pipeline (v8.0.0; 10X Genomics). Filtered feature bc matrix output from CellRanger was used for the standard Seurat analysis (v5.1.0) (98). Quality control was first conducted by removing cells with less than 200 genes, more than 10% UMI mapped to mitochondrial genes and hemoglobin genes. SCTransform package (v0.4.1) was then used to the filtered cells for normalization, PCA dimensionality reduction, and UMAP embedding to generate the cell clusters. WT and *mdx* myeloid cell population datasets from Farahat et al. (38) and Coulis et al. (33) were integrated using Seurat with anchor-based RPCA integration of IntegrateLayers function. Comparative cell-cell interaction inference analysis in *DysfA6* was conducted using CellChat (version 2.1.2). Briefly, fibroblast and macrophage subtype Seurat objects were loaded and merged as a single CellChat object. Significant signaling networks between FAPs and specific macrophage subtypes in *DysfA6* were identified in CellChat, as described previously (99).

### Isolated myofiber bulk RNA-seq.

Quadriceps were removed from *Dysf* and *DysfA6* female mice (*n* = 2 each, 4 mice total) and digested in 6 mg/mL collagenase type II (LS004177, Worthington) solution in 1:5 weight to volume ratio at 37°C for 2.5 hours, then filtered through a 70 μm nylon filter. Remaining tissues on the filter were washed with DPBS with calcium and magnesium (MT21030CV, Thermo Fisher Scientific), and then filtered through a 100 μm nylon filter. RNA was isolated using TRIzol Reagent (15596018, Thermo Fisher Scientific) followed by Aurum Total RNA Mini Kit (7326820, Bio-Rad). RNA quality and quantity were assessed by Bioanalyzer RNA Pico chip and Qubit RNA HS assay kit, respectively. Multiplexed libraries were sequenced on a NovaSeq X Plus using single-end 50-nt mode. Sequenced raw reads were processed through FASTQC (v0.11.5) (https://github.com/s-andrews/fastqc), trimmed with trimmomatic (v0.36) (https://github.com/usadellab/trimmomatic), and aligned to the mouse reference genome (mm10) using STAR (v2.5.2) (https://github.com/alexdobin/STAR). Raw read counts were quantified with HTSeq (https://github.com/simon-anders/htseq), then processed and normalized using vst function in DESeq2 (v1.44.0) package in RStudio (https://docs.posit.co/ide/user/#rstudio-ide-oss-downloads). Raw read counts from bulk RNA-seq of WT myofibers were obtained from Salamone et al. (49) and merged with counts from *Dysf* and *DysfA6* myofibers. Significantly upregulated genes were defined as having a *P* value of less than 0.05 and log_2_(fold change) greater than 0. Kyoto Encyclopedia of Genes and Genomes (KEGG) enrichment analysis was performed with significantly upregulated genes from each model using the enrichKEGG function in the clusterProfiler (v4.12.6) package in RStudio, filtered for Cell Processes KEGG pathways with a *P* value of less than 0.05. Heatmap was visualized from vst normalized values, scaled by row, using pheatmap (v1.0.12) package in RStudio (Supplemental Figure 5, B and C).

### Macrophage isolation and culture.

Bone marrow cells were flushed from isolated femurs and tibias of 12- to 16-week-old WT mice using prewarmed DMEM (10-014-CV, Corning). Cells were treated with RBC lysis buffer (00-4333-57, Thermo Fisher Scientific) and filtered through a 40 μm nylon filter, and plated in macrophage culture media (DMEM with 10% fetal bovine serum, 1% sodium pyruvate [25-000-CL, Corning], 1% L-glutamine [25030081, Thermo Fisher Scientific], and 1% penicillin-streptomycin with 10 ng/mL M-CSF [576408, BioLegend]) at 37°C, 5% CO_2_. Four days after isolation and plating, half the media was supplemented with 10 ng/mL M-CSF. Seven days after the isolation, full media change with M-CSF was conducted every 3–4 days. Cell were used within 1–2 weeks.

### Ex vivo phagocytosis assay.

Twenty-four hours before the assay, 500,000 macrophages were plated on MatTek dishes (P35G-1.5-14-C, MatTek) in macrophage culture media and cultured at 37°C 5% CO_2_. One to 2 hours after plating, 1 mL of macrophage culture media was added to the plate to prevent evaporation. On the day of the assay, FDB myofibers were isolated as indicated above using collagenase. Seventy-five minutes after collagenase digestion, the myofibers were triturated in 1× Ringer’s with 1× CellEvent Caspase3/7 Detection Green (C10432, Thermo Fisher Scientific), 1× pHRodo Red AM (P35372, Thermo Fisher Scientific), and Hoechst. Isolated FDBs were subsequently incubated in the dye solution for 30 minutes at 37°C, 10% CO_2_. The stained myofibers were then washed with 1× Ringer’s solution and resuspended in macrophage culture media. Macrophages, isolated and cultured as above, were stained with LysoTracker Deep Red (L12492, Thermo Fisher Scientific) for 30 minutes at 37°C, 5% CO_2_ and subsequently washed 3 times to remove the dye solution. The labeled myofibers were then incubated with labeled macrophages and incubated at 37°C, 5% CO_2_ for 2 hours. After incubation, CASP3^+^ myofibers (10–15 per mouse) with at least one pHRodo^+^ macrophage were selected for imaging on a Keyence BZ-X810 microscope. Phagocytosis was quantified by dividing the number of pHRodo^+^ macrophages by the total number of macrophages on or immediately adjacent to each CASP3^+^ myofiber. Representative images were acquired on a Nikon A1R laser-scanning confocal microscope with 40× Apo lambda 0.95 NA objective with 2.5× zoom.

### Sample preparation for proteomic analysis and LC-MS/MS analysis.

Skeletal muscle samples were processed as previously described (54, 100, 101). Fractions were subsequently subjected to enzymatic digestion overnight (16 hours) at 37°C with trypsin (1:100 enzyme to protein ratio) using a filter-aided sample preparation (FASP) approach as previously described (102) and desalted during Evotip loading. Analysis used MSFragger v4.1 via FragPipe v22.0 (MSFragger: https://msfragger.nesvilab.org/), (https://fragpipe.nesvilab.org/). Cellular, soluable ECM, and insoluable ECM fractions were searched separately and merged after database searching. Results were filtered to 1% FDR at the peptide and protein level. The raw intensity of each protein from each fraction was analyzed using MetaboAnalyst 6.0 (103). Differentially expressed proteins defined as having an FDR of less than 0.05 and log_2_(fold change) greater than 0.5 or less than –0.5 were plotted as volcano plots in RStudio using ggplot2.

### Decellularization.

Decellularization of quadriceps sections was performed as described in Long et al. (53).

### Incubating macrophages on decellularized myoscaffolds.

Decellularized myoscaffolds generated as indicated above were assembled in chamber slides (CCS-2, MatTek) and were sterilized in DMEM with 1% penicillin-streptomycin and 1% Amphotericin B (A2942-20ML, Sigma-Aldrich) for 24 hours at 37°C, 5% CO_2_. On the following day, 500,000 macrophages were seeded onto the sterilized myoscaffolds and cultured in macrophage culture media with 10 ng/mL of M-CSF for 24 hours. After 24 hours, the seeded myoscaffolds were fixed in 4% PFA and processed for IFM as indicated above. Myoscaffolds were sourced from *n* = 3 mice per genotype, and the macrophages were sourced from *n* = 3 WT mice.

### Quantification of macrophage ECM localization and morphology.

Five representative ×40 *Z*-stack images of each seeded myoscaffold were acquired per condition. A fixed range of 30 μm and a pitch of 0.5 μm was utilized, with a total of 61 images taken per *Z*-stack. To determine macrophage localization, nuclei were counted in the Hoechst channel. Total number of nuclei, per *Z*-stack, was counted via analysis of the entire *Z*-stack (61/61 images) using the Keyence BZ-X800 analyzer software. In order to determine the localization to the glass and lower part of the ECM, the lower 33% of the *Z*-stack (20/61 images) was analyzed. Through subtraction of the nuclei quantified from the bottom 33% of the *Z*-stack from the full *Z*-stack, the number of macrophages localized to the middle and top of the matrix (41/61 images) was calculated. To quantify the circularity of the macrophages on the ECM, all the macrophages in each representative image were traced and the circularity was quantified using FIJI (NIH).

### Recombinant ANXA1 and ANXA2 treatment of macrophages.

Macrophages (500,000 per well) were plated in 24-well plates in macrophage culture media without M-CSF. After 24 hours, 1 mg/mL recombinant ANXA1 (230-00601-50, RayBiotech) and ANXA2 (230-30023-50, RayBiotech) were diluted to 5 μg/mL in macrophage culture media and subsequently added to plated macrophages for 3 hours. After the treatment, the cells were harvested for downstream gene expression analysis.

### Quantitative real-time PCR.

RNA was extracted with frozen macrophage cell pellets using TRIzol with application of qScript cDNA SuperMix (95048-025, VWR). Quantitative real-time PCR was performed using iTaq Universal SYBR Green Supermix (1725124, Bio-Rad Laboratories). *18S* was used as loading control. Fold change in expression was calculated using the ΔΔCt method. *Csf1*: 5′-GCCCTTCTTCGACATGGCT-3′, Reverse 5′-CCTTCAGGTGTCCATTCCCA-3′. *18S*: Forward 5′-GTAACCCGTTGAACCCCATT-3′, Reverse 5′-CCATCCAATCGGTAGTAGCG-3′.

### Statistics.

Statistical analyses were performed using Prism software v10 (GraphPad). When comparing 2 groups, 2-tailed Student’s *t* test with Welch’s correction (unequal variances) was used. When comparing 3 or more groups for only 1 variable, 1-way ANOVA with Tukey’s multiple-comparison test was used. When comparing data groups for more than 1 related variable, 2-way ANOVA was performed. A *P* value of less than or equal to 0.05 was considered significant. Data are presented as mean ± SEM.

### Study approval.

All procedures using mice followed the NIH *Guide for the Care and Use of Laboratory Animals* (National Academies Press, 2011) and were approved by Northwestern University’s Institutional Animal Care and Use Committee.

### Data availability.

scRNA-seq and myofiber bulk RNA-seq data are deposited in NCBI’s Gene Expression Omnibus data repository under accession numbers GSE328601 and GSE328583, respectively. The MS ECM proteomics data have been deposited to the ProteomeXchange Consortium via the PRIDE (104) partner repository with the dataset identifier PXD077779.

## Author contributions

GL, DD, JMK, and ARD performed fluorescence imaging and analysis. MH, LAV, GL, and AJF performed mouse husbandry and injections. GL, LAV, DD, and ARD performed muscle isolations and related immunoblots. GL, CL, ABW, and ARD performed the single-cell and bulk RNA sequencing analysis. GL, MM, DCG, and KCH performed and analyzed quantitative proteomics datasets. GL, LAV, DD, JMK, and ARD performed the EBD and creatine kinase studies. LAV, DD, ARK, and ARD performed the mouse physiological assessments. MTD and ARD performed the laser injury and analysis. PGTP and DD performed histological stains. CL and EBT provided the protocols for bone marrow–derived macrophage isolation and efferocytosis assay. GL, AJF, and DD performed macrophage isolation and the ex vivo phagocytosis assay. GL and AML performed decellularization and seeding of myoscaffolds and in vitro macrophage assays. GL, EMM, RHC, and ARD conceived and designed the studies, analyzed the data, and wrote the manuscript.

## Conflict of interest

Northwestern University filed provisional patents (62/783,619 and 63/309,925) on behalf of ARD and EMM. EMM is or has been a consultant to Amgen, AstraZeneca, Cytokinetics, PepGen, Pfizer, Tenaya Therapeutics, and Novartis and is the CEO of Ikaika Therapeutics. ARD is the CSO of Ikaika Therapeutics.

## Funding support

This work is in part the result of NIH funding and is subject to the NIH Public Access Policy. Through acceptance of this federal funding, the NIH has been given a right to make the work publicly available in PubMed Central.

- NIH grants NS047726, HD119693, and AR052646.
- Parent Project Muscular Dystrophy.
- Muscular Dystrophy Association.
- Lakeside Discovery.
- NIH grant P30CA046934 (University of Colorado Cancer Center Support Grant).
- NIH grant 1S10OD025120 (to the Northwestern University NUseq facility core).

## Supplementary Material

Supplemental data

Unedited blot and gel images

Supporting data values

## Version 1 07/06/2026

In-Press Preview

## Version 2 08/10/2026

Electronic publication
