# Supplementary material for: Excess muscle plasma membrane leak disrupts ECM content and shifts macrophage-mediated muscle repair
Source: JCI Insight. 2026 Jun 23;11(15):e205137. doi: 10.1172/jci.insight.205137 (PMC13464014; doi:10.1172/jci.insight.205137)
Supplement: Supplementary file 1 [file jciinsight-11-205137-s237.pdf]

SUPPLEMENTAL FIGURES AND LEGENDS FOR LEE ET AL.

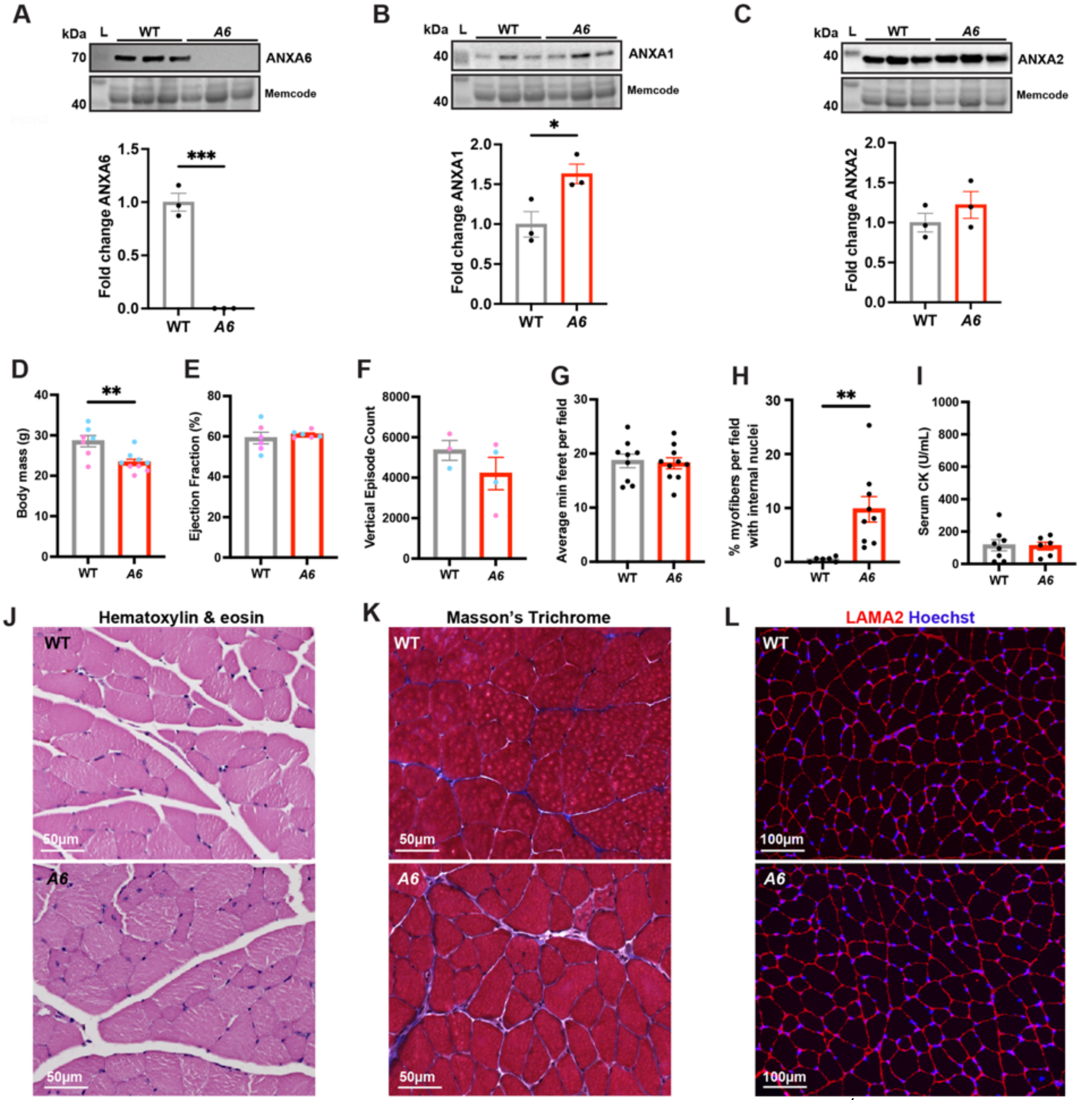

Supplemental Figure 1. Minimal histopathological changes in *Anxa6*$^{-/-}$ *(A6)* mice. (**A**) Immunoblot of WT and *A6* muscle lysates confirms loss of ANXA6 protein. (**B**) Immunoblotting of WT and *A6* muscle shows increased ANXA1. (**C**) Immunoblot for ANXA2 in WT and A6 muscle shows comparable amounts. (**D**) Reduced total body mass in *A6* mice compared to WT controls. (**E**) Ejection fraction % was similar between A6 and WT mice. (**F**) Mouse vertical episode activity was similar. Male (blue dots), female (pink dots). n=5-6 mice per genotype. (**G**) Myofiber size plotted as average minimum feret diameter per field was similar between WT and *A6* mice. (**H**) *A6* muscle had a significantly increased percentage of myofibers per field with internal nuclei. (**I**) Similar serum creatine kinase (CK) between WT and *A6* mice. (**J**) Representative hematoxylin and eosin images of quadriceps muscle from WT and *A6* mice. (**K**) Representative Masson's Trichrome images of quadriceps muscle from WT and *A6* mice. (**L**) LAMA2 staining outlines myofibers. Hoechst marks nuclei. Two-tailed Student's t-test, *$p < 0.05$, **$p < 0.01$.

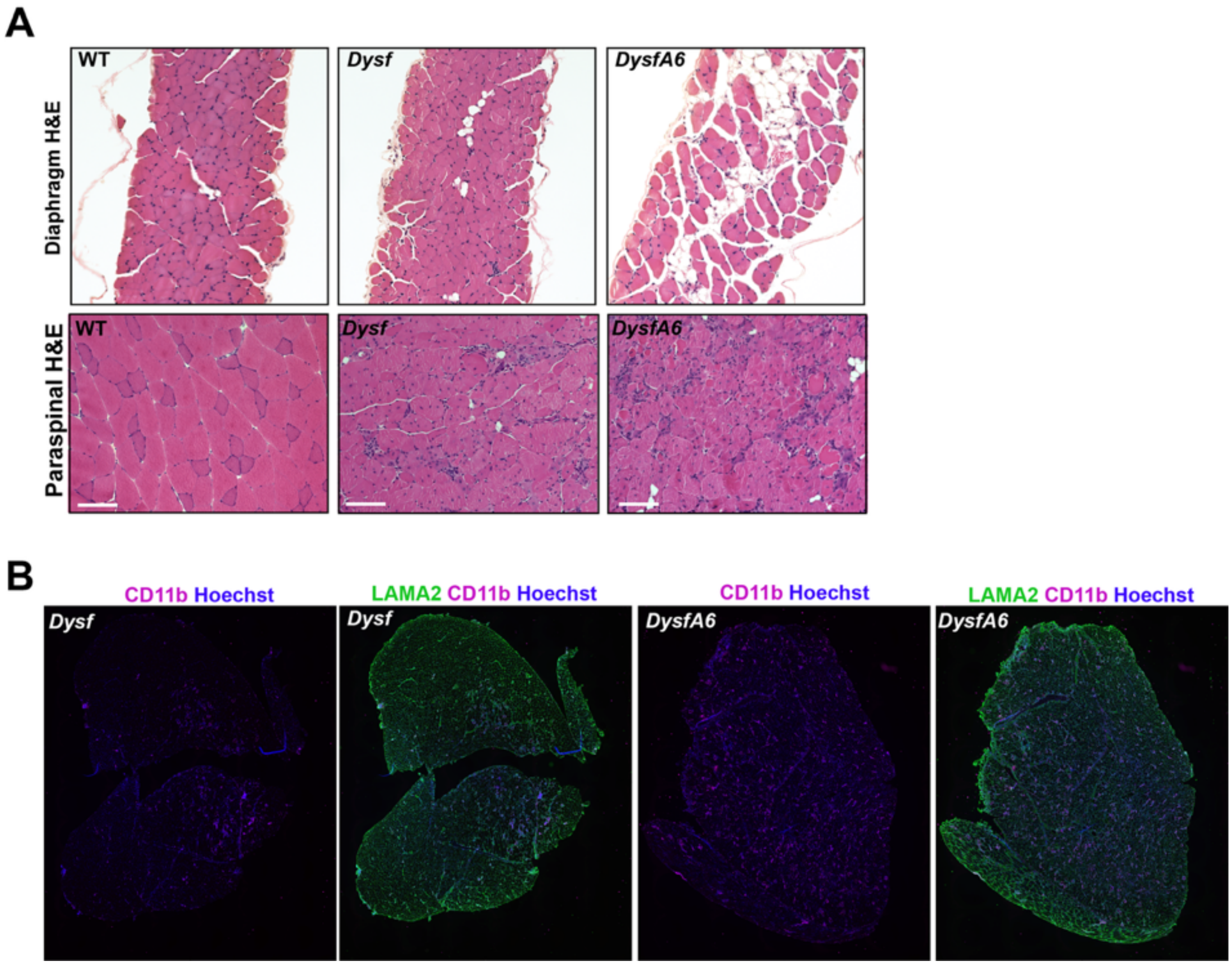

Supplemental Figure 2. Increased disease pathology across multiple *DysfA6* muscle groups compared to *Dysf* muscle. (**A**) Hematoxylin and Eosin staining showed greater fibrofatty and immune infiltrate in *DysfA6* diaphragm and paraspinal spinal muscles compared to *Dysf* muscle. (**B**) Immunofluorescence imaging of whole quadriceps muscles demonstrating increased CD11b cellular infiltrate throughout the *DysfA6* muscle compared to *Dysf*.

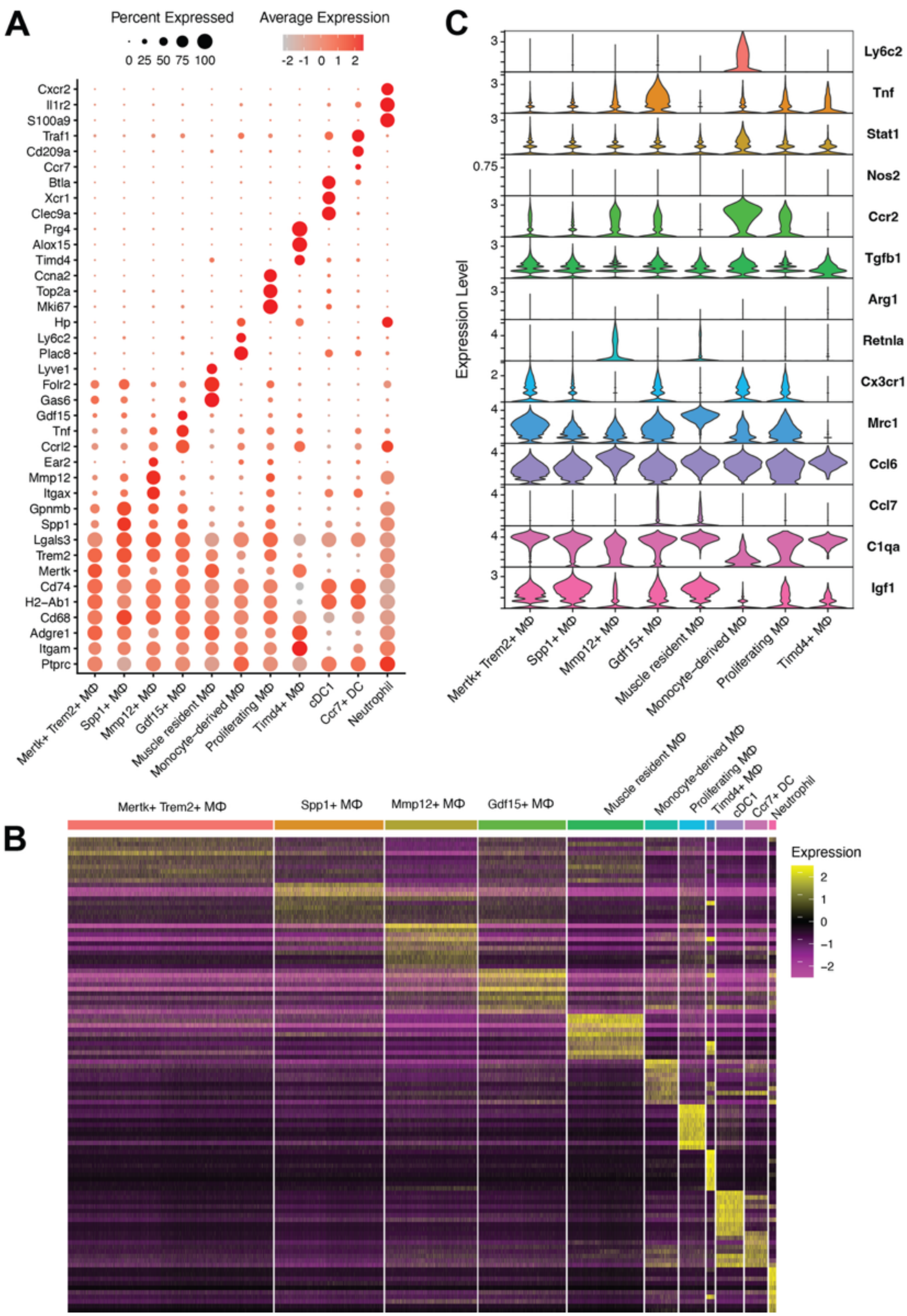

Supplemental Figure 3. M1 and M2 marker genes distribute across many macrophage subpopulations in *Dysf/DysfA6* skeletal muscle. (**A**) Key marker genes used to identify different myeloid cell subpopulations in *Dysf* and *DysfA6* muscle scRNA-seq data. (**B**) Heatmap of top 10 differentially expressed genes from each myeloid cell types in *Dysf* and *DystA6* scRNA-seq data. (**C**) Violin plots illustrating canonical M1 and M2 macrophage marker gene expression and broad distribution across macrophage subtypes in *Dysf* and *DysfA6*.

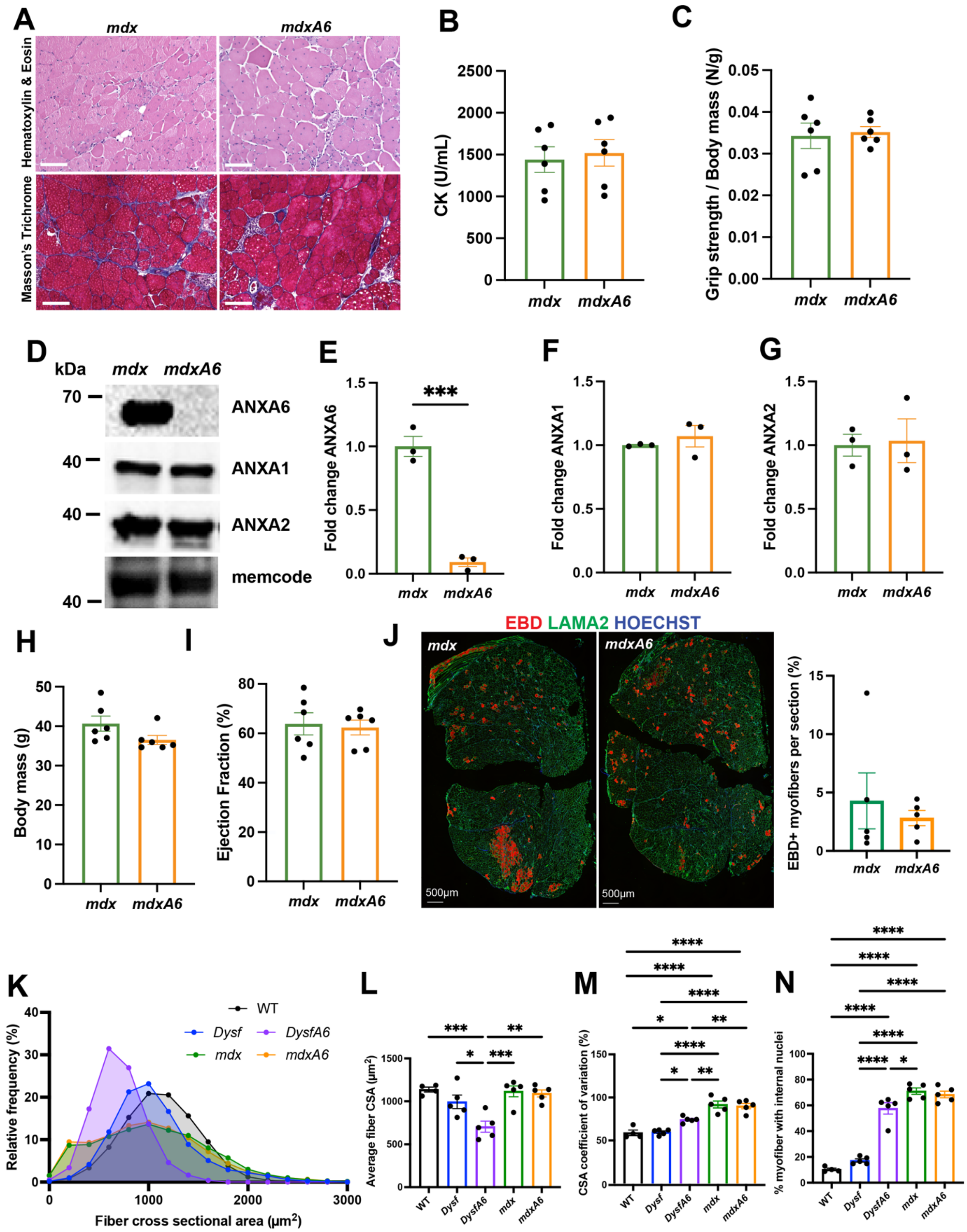

Supplemental Figure 4. *mdxA6* and *mdx* mice show similar muscle pathology and function. (**A**) Representative Hematoxylin & Eosin and Masson's Trichrome images of quadriceps muscle from *mdx* and *mdxA6* mice. (**B**) Serum creatine kinase (CK) was unchanged between *mdx* and *mdxA6* mice. (**C**) Normalized forelimb grip strength was similar between *mdx* and *mdxA6* mice (n=6 mice per genotype, all males). (**D**-**G**) Immunoblot

quantification of *mdx* and *mdxA6* muscle lysates confirmed loss of ANXA6 protein with no significant change in ANXA1 and ANXA2. (**H** and **I**) Body mass and cardiac ejection fraction (%) were similar between *mdx* and *mdxA6* mice. (**J**) Immunofluorescence image of full quadriceps muscle cross-section isolated from *mdx* and *mdxA6* mice injected with EBD demonstrated similar number of EBD (red) positive myofibers in both genotypes. LAMA2 (green) outlined the muscle section (n=5 mice per genotype, 2 sections per mouse). (**K**) Relative frequency of myofiber CSA is similar between *mdx* and *mdxA6*, along with (**L**) average CSA, (**M**) CSA variation, and (**N**) percentage of myofibers with internal myonuclei. Two-tailed Student's t-test for comparison between two groups, one-way ANOVA for comparison across 5 groups, *p<0.05, **p<0.01, ***p<0.005, ****p<0.001. Scale bar 100μm.

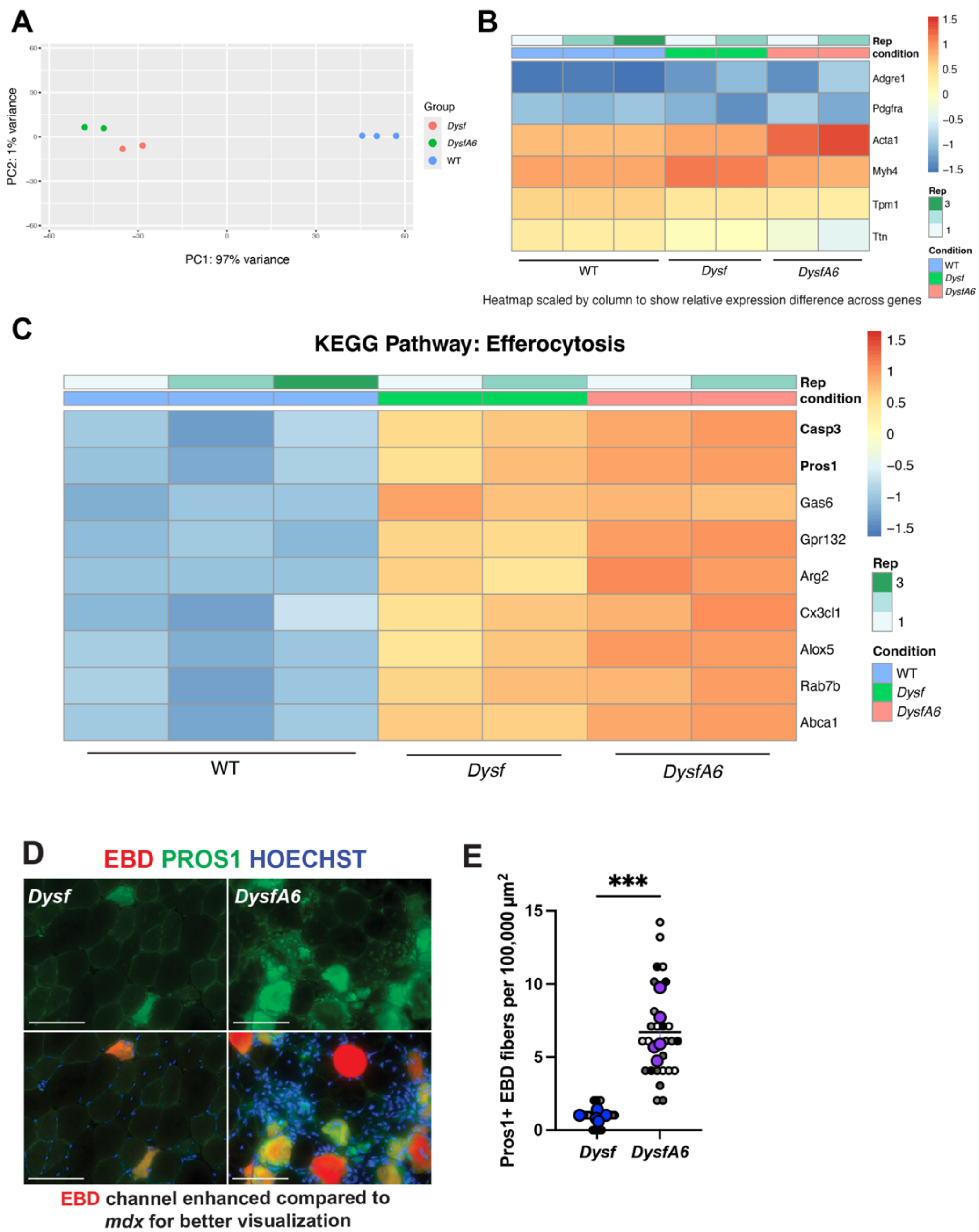

Supplemental Figure 5. Defective membrane repair increases efferocytosis gene expression in *DysfA6* myofibers. (**A**) The myofiber fraction of muscle was isolated and bulk RNA-seq was completed and analyzed. The WT sequence data was from (49). Principal Component Analysis (PCA) demonstrates clustering of samples from each genotype. (**B**) Higher expression of sarcomeric genes than mononuclear cell markers (*Adgre1* and *Pdgfra*) within each sample confirmed enrichment of myofiber fractions. (**C**) Heatmap of upregulated efferocytosis genes in WT, *Dysf* and *DysfA6* myofiber fraction showed sequential increase in expression: WT < *Dysf* < *DysfA6.* (**D**) Representative IFM images of EBD-injected muscle, demonstrated distinct patterns of PROS1 expression in *Dysf* and *DysfA6* muscle EBD (red), PROS1 (green), nuclei (blue). (**E**) Quantification of PROS1+/EBD+ fibers demonstrated that having more impaired repair leads to more PROS1+/EBD+ fibers. Two-tailed Student's t-test, ****p < 0.001. Scale bar 100µm.

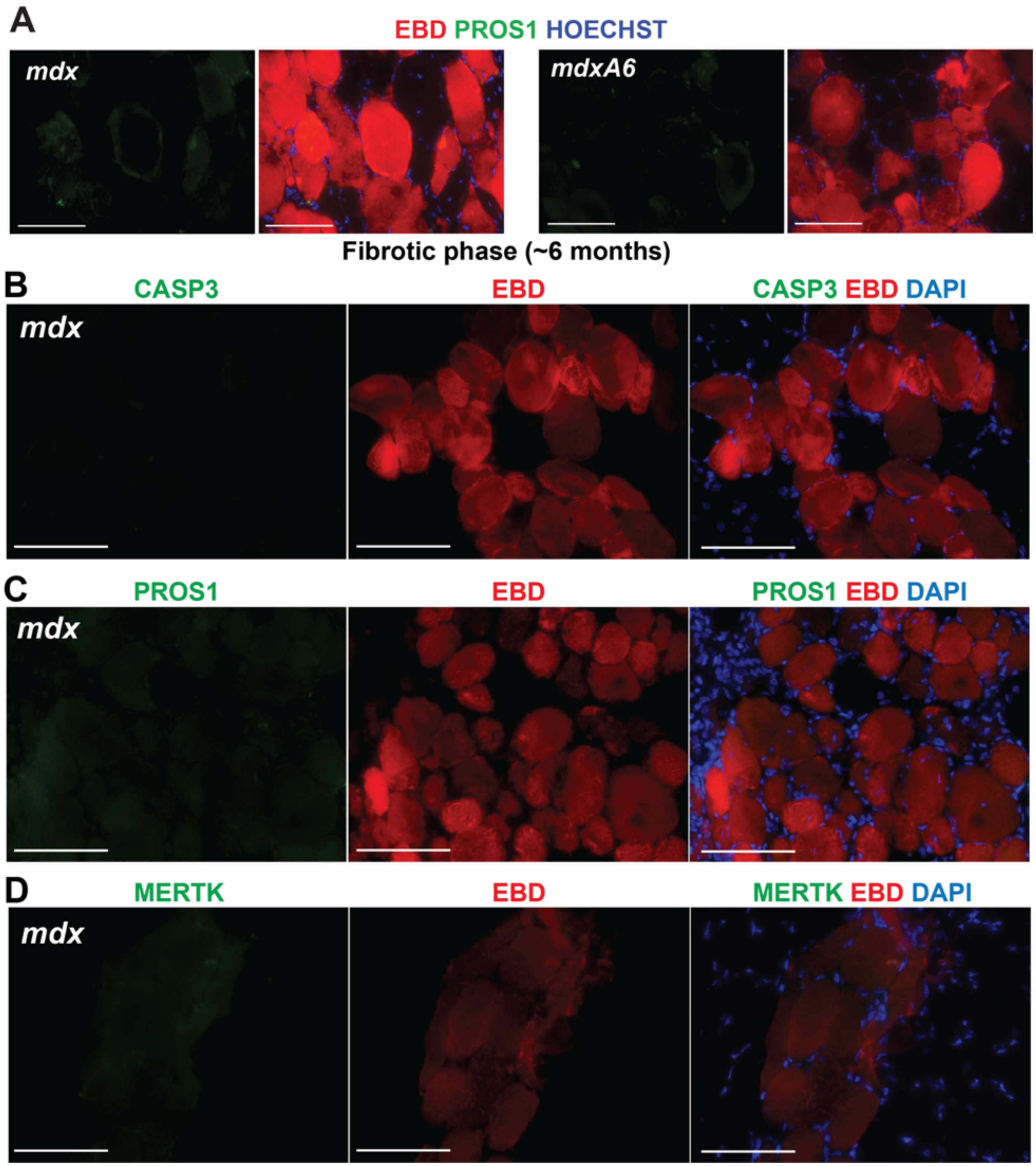

Supplemental Figure 6. Lower MERTK, CASP3 and PROS1 expression in EBD+ fibers in *mdx and mdxA6* muscle compared to *Dysf* and *DysfA6*. (**A**) Strongly EBD positive (red) myofibers in *mdx* and *mdxA6* muscle were less PROS1 positive at fibrotic phase of 6 months. (**B** and **C**) Four-week, highly inflamed *mdx* muscle displayed EBD positive myofibers with low to no CASP3 or PROS1, and MERK expression. Scale bar 100μm.

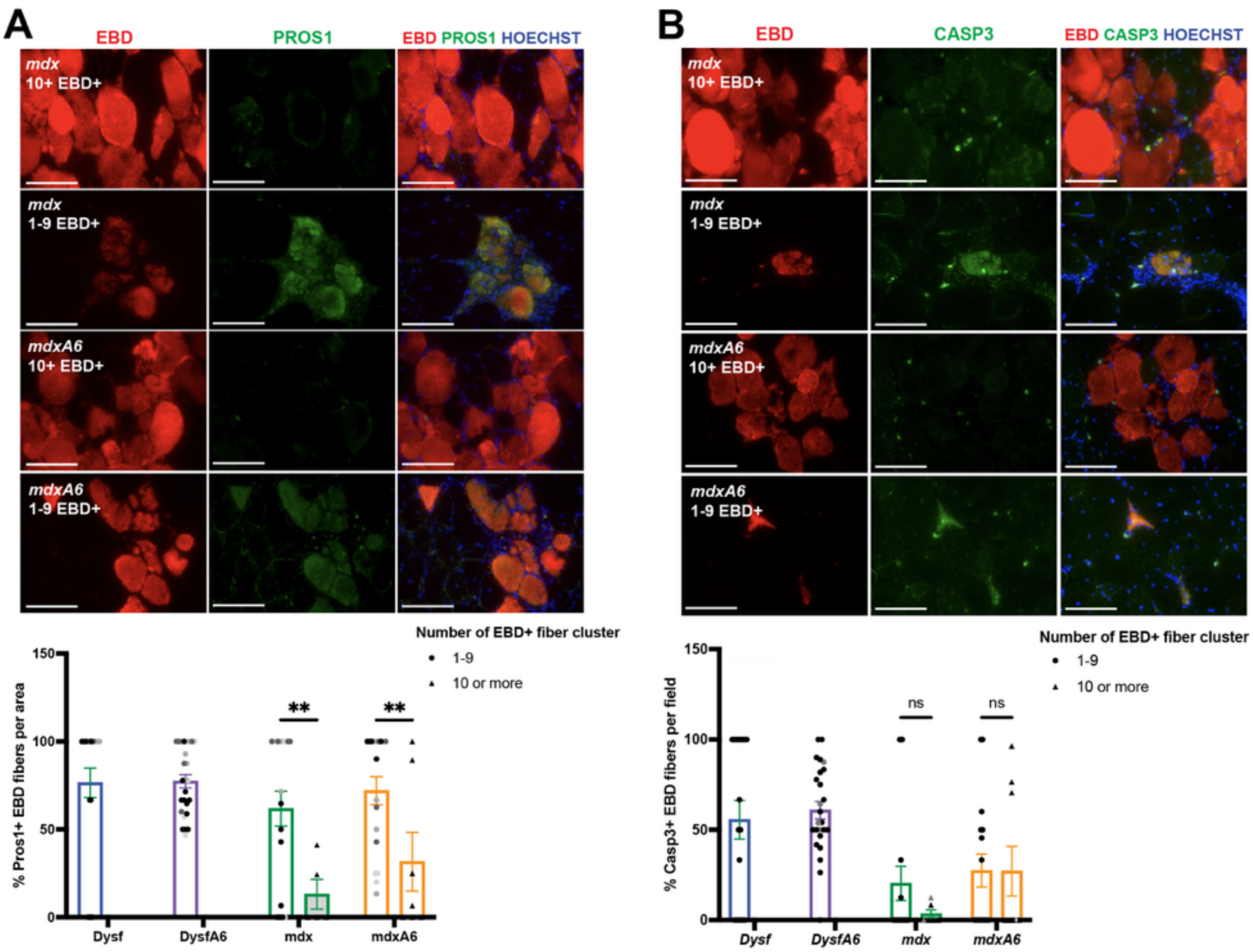

Supplemental Figure 7. Lack of activation of cleaved caspase-3 and protein S in areas of large lesion in *mdx* and *mdxA6* muscle. (**A** and **B**) Areas of large lesions with more than 10 EBD+ myofibers had fewer PROS1+/EBD+ or CASP3+/EBD+ myofibers in both *mdx* and *mdxA6*. Large EBD+ clusters were not seen in *Dysf* and *DysfA6* muscles; all EBD+ fibers were in clusters of 1-9 EBD+ fibers per clusters (n=5 mouse per genotype with 2 sections per mouse males only). Two-way ANOVA, **p<0.01. Scale bar 100µm.

Supplemental Table 1. Antibodies used in this study.

Antibody	Source	Catalog #	Dilution
Annexin A1	Thermo Fisher Scientific	PA5-22266	1:100
Annexin A2	BD Bioscience	610068	1:100
Annexin A6	Abcam	ab199422	1:100
Laminin-2	Sigma-Aldrich	L0663	1:100
PDGFRa	R&D Systems	AF1062	1:40
CD11b	Thermo Fisher Scientific	17-0112-82	1:100
CD11b	Thermo Fisher Scientific	14-0112-82	1:100
PROS1	Novus Bio	NB431506	1:100
Cleaved caspase-3 (CASP3)	Cell Signaling	9661T	1:100
MERTK	Thermo Fisher Scientific	14-5751-81	1:100
MERTK	R&D systems	AF591-SP	1:100
F4/80	Abcam	ab6640	1:100
Alexa Fluor 647 donkey anti-rat	Thermo Fisher Scientific	A78947	1:2500
Alexa Fluor 594 donkey anti-rat	Thermo Fisher Scientific	A11007	1:2500
Alexa Fluor 488 donkey anti-rat	Thermo Fisher Scientific	A11006	1:2500
Alexa Fluor 488 donkey anti-rabbit	Thermo Fisher Scientific	A11008	1:2500
Alexa Fluor 647 donkey anti-rabbit	Thermo Fisher Scientific	A31573	1:2500
Alexa Fluor 594 donkey anti-rabbit	Thermo Fisher Scientific	A11012	1:2500
Alexa Fluor 594 donkey anti-goat	Thermo Fisher Scientific	A11058	1:2500
Hoechst 33342	Thermo Fisher Scientific	H3570	1:25,000
Goat anti-Rat IgG HRP	Thermo Fisher Scientific	31470	1:2500
Goat anti-rabbit IgG HRP	Thermo Fisher Scientific	NC9734651	1:2500
Donkey anti-Goat IgG HRP	Thermo Fisher Scientific	A15999	1:2500
