# Supplementary material for: Excess muscle plasma membrane leak disrupts ECM content and shifts macrophage-mediated muscle repair
Source: JCI Insight. 2026 Jun 23;11(15):e205137. doi: 10.1172/jci.insight.205137 (PMC13464014; doi:10.1172/jci.insight.205137)
Supplement: Supplementary file 2 [file jciinsight-11-205137-s238.pdf]

Figure 7 Full blots

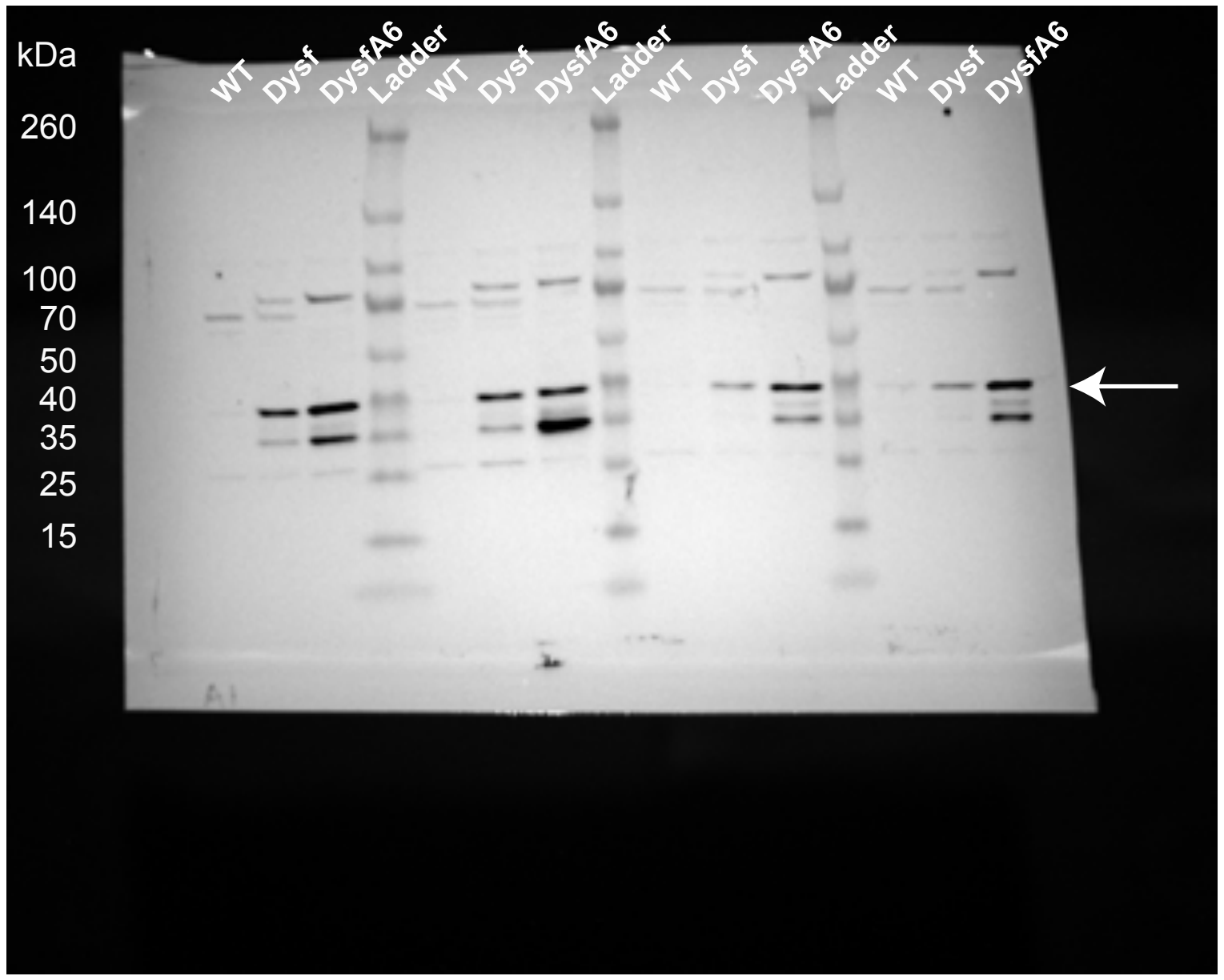

ANXA1

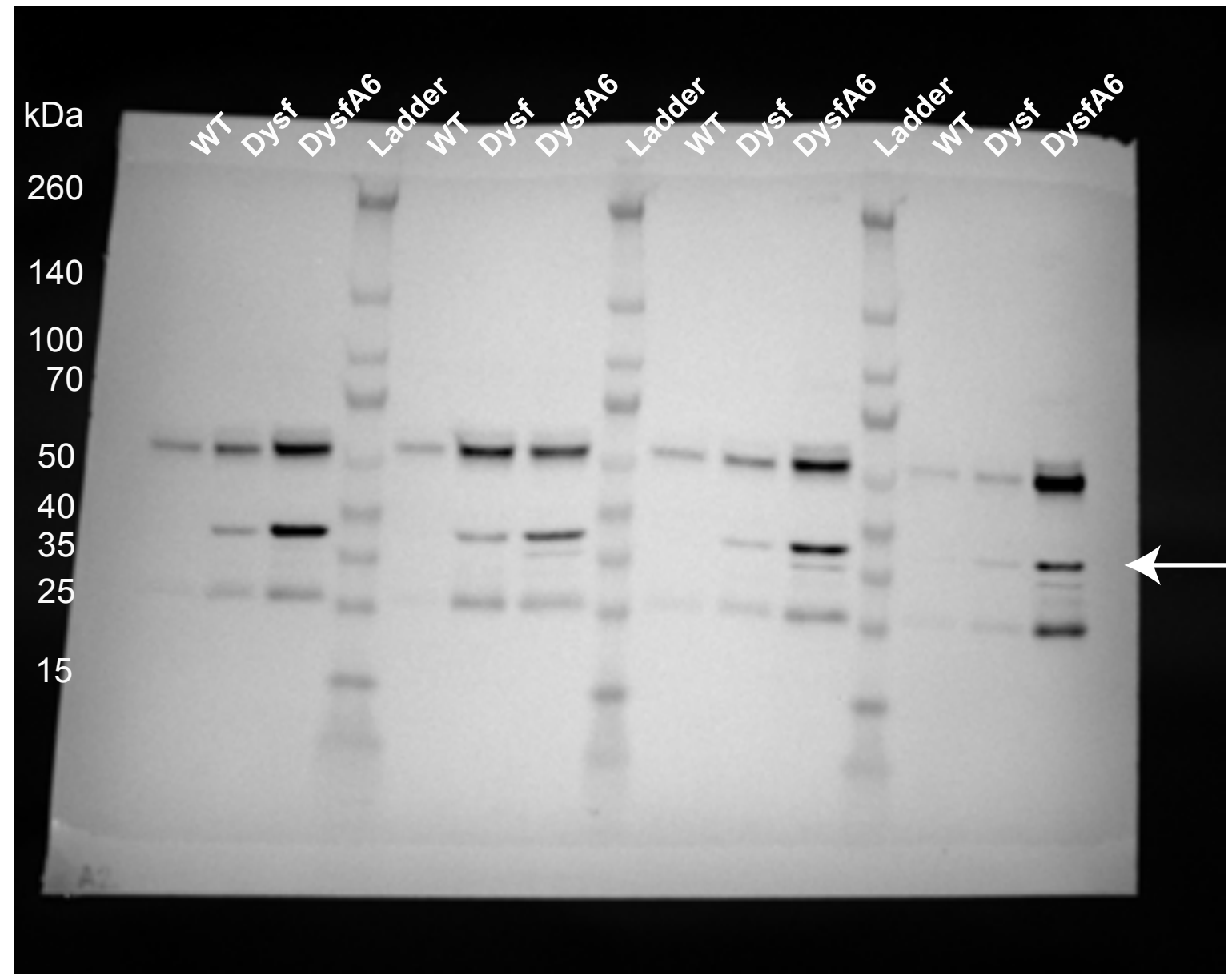

ANXA2

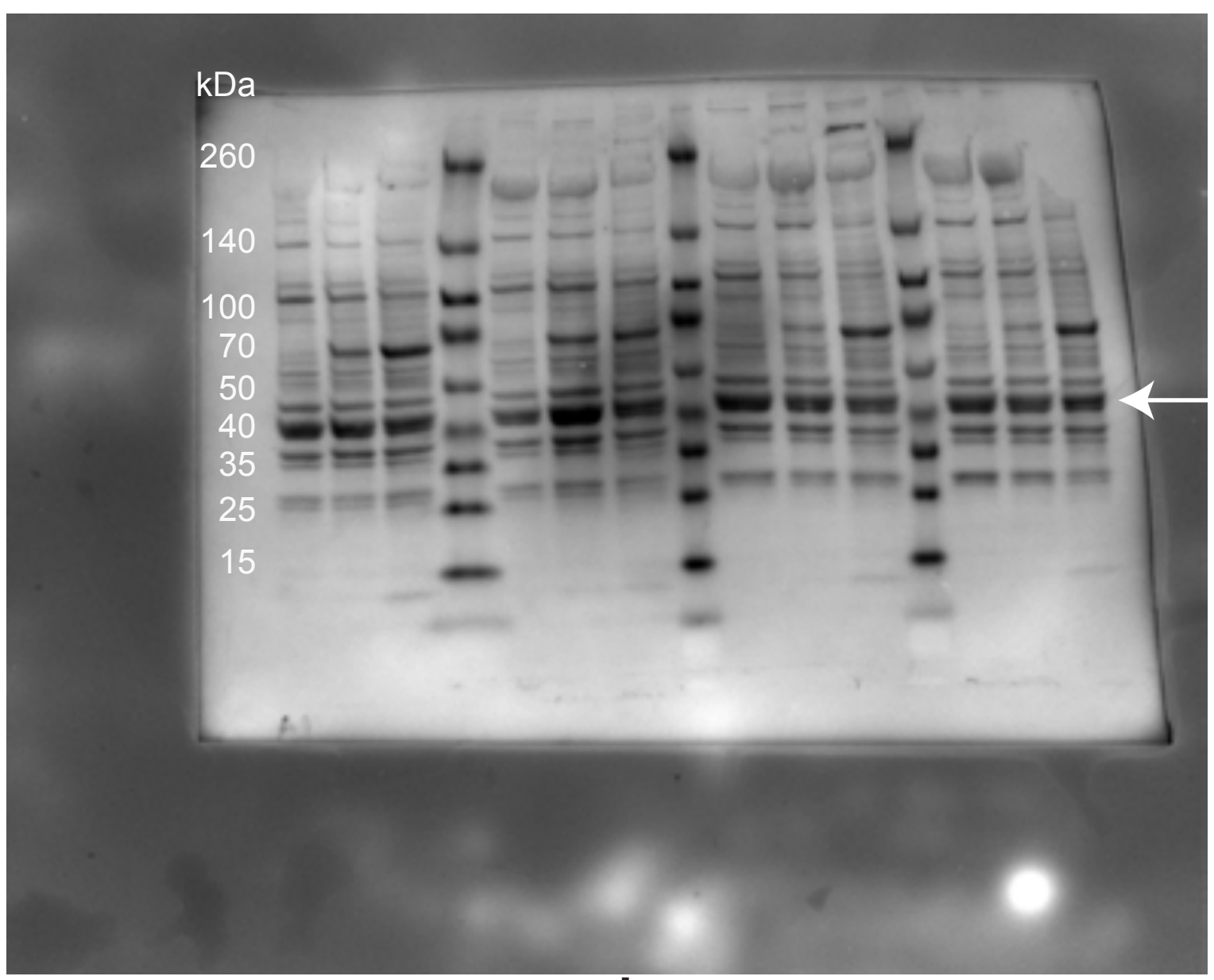

memcode

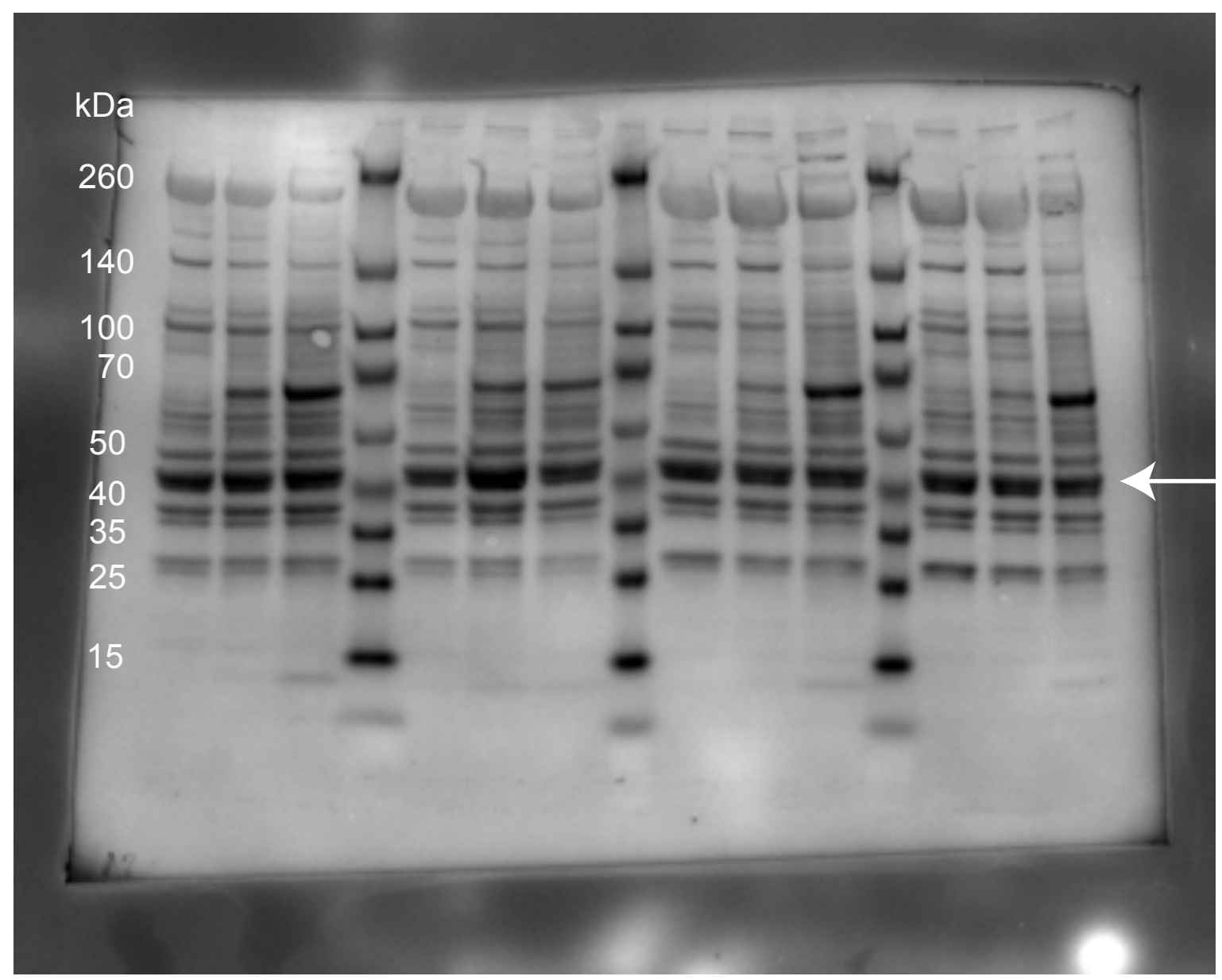

memcode

Supplemental Figure 1 Full blots

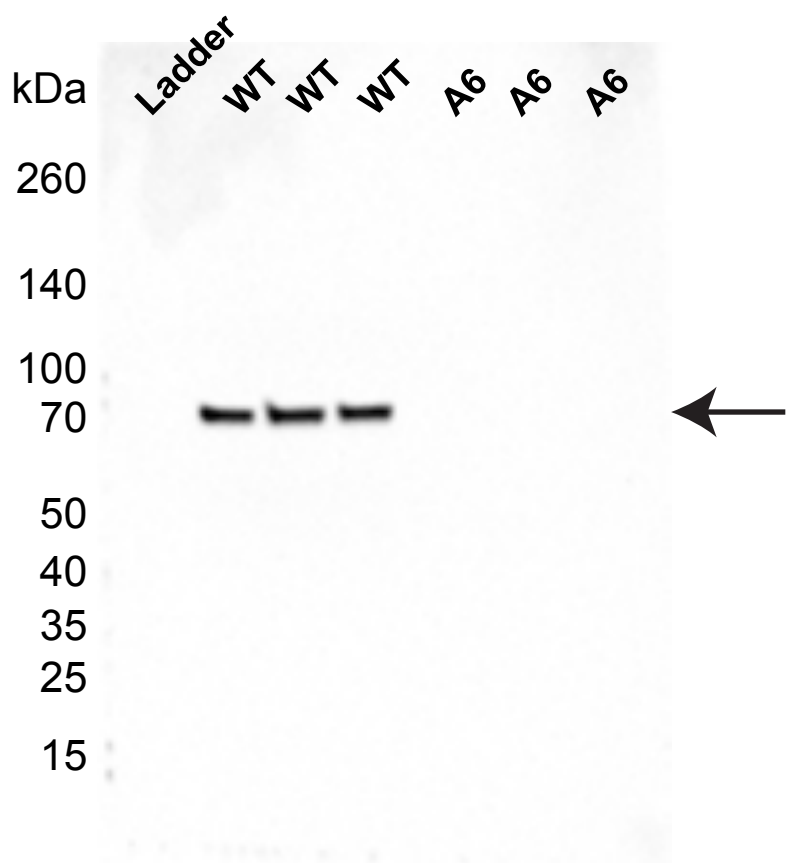

ANXA6

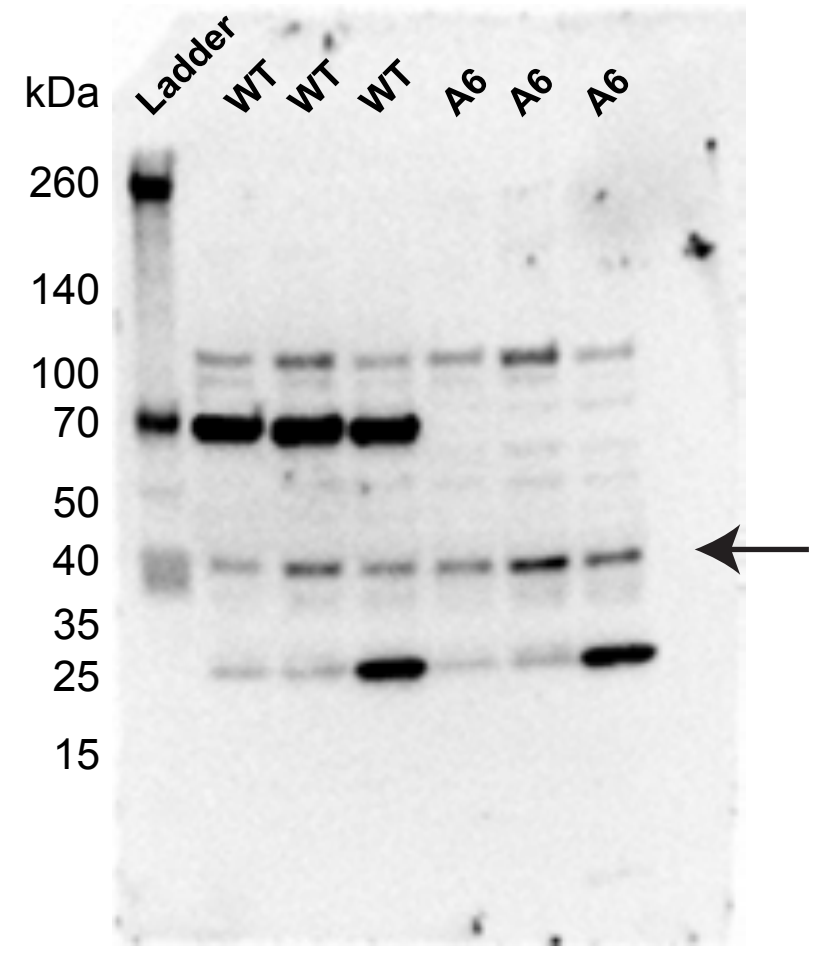

ANXA1

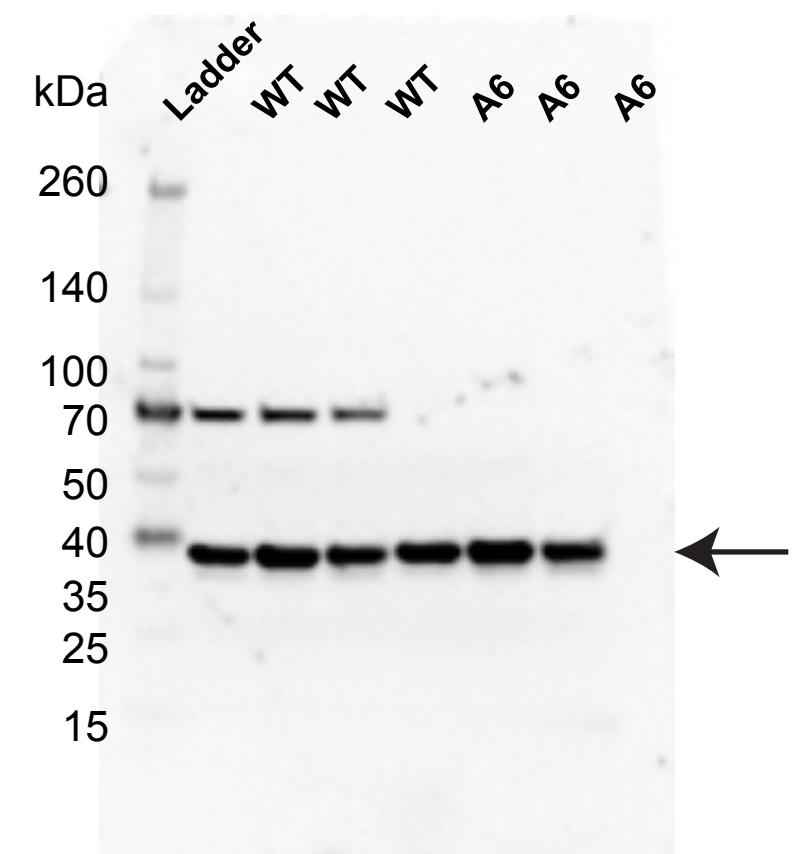

ANXA2

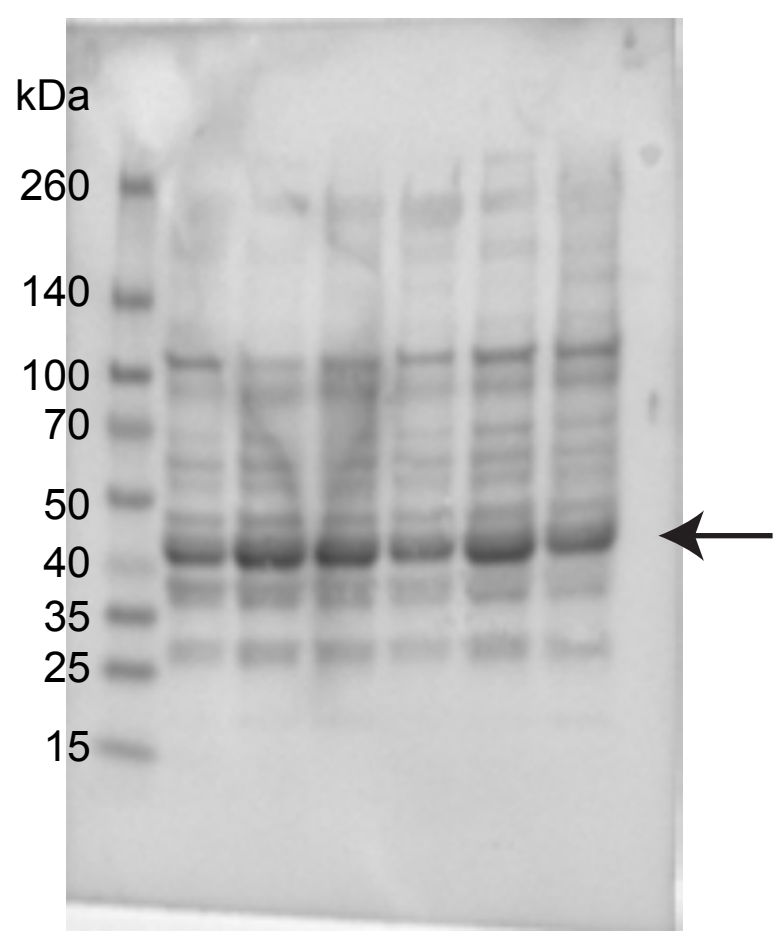

memcode

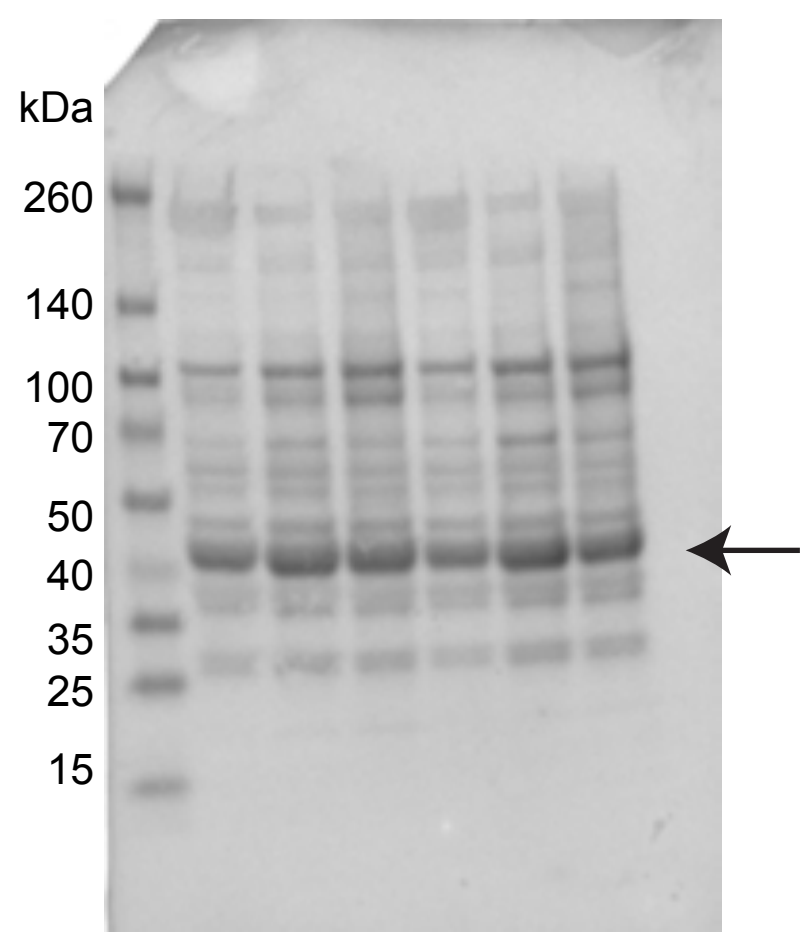

memcode

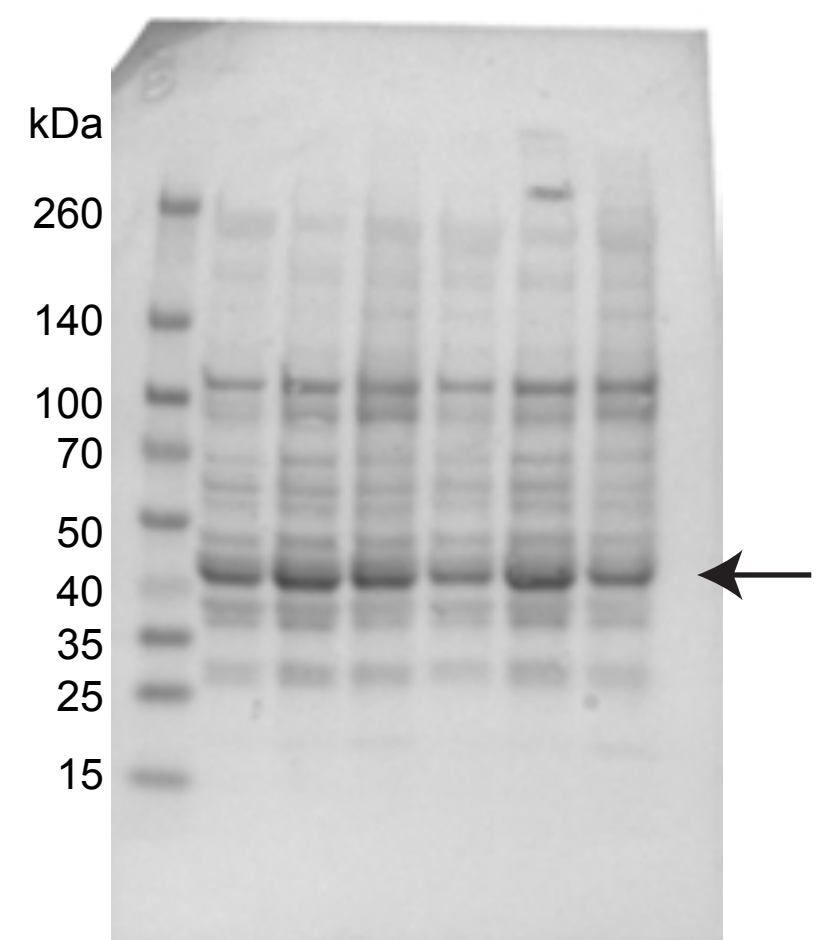

memcode

Supplemental Figure 4 Full blots

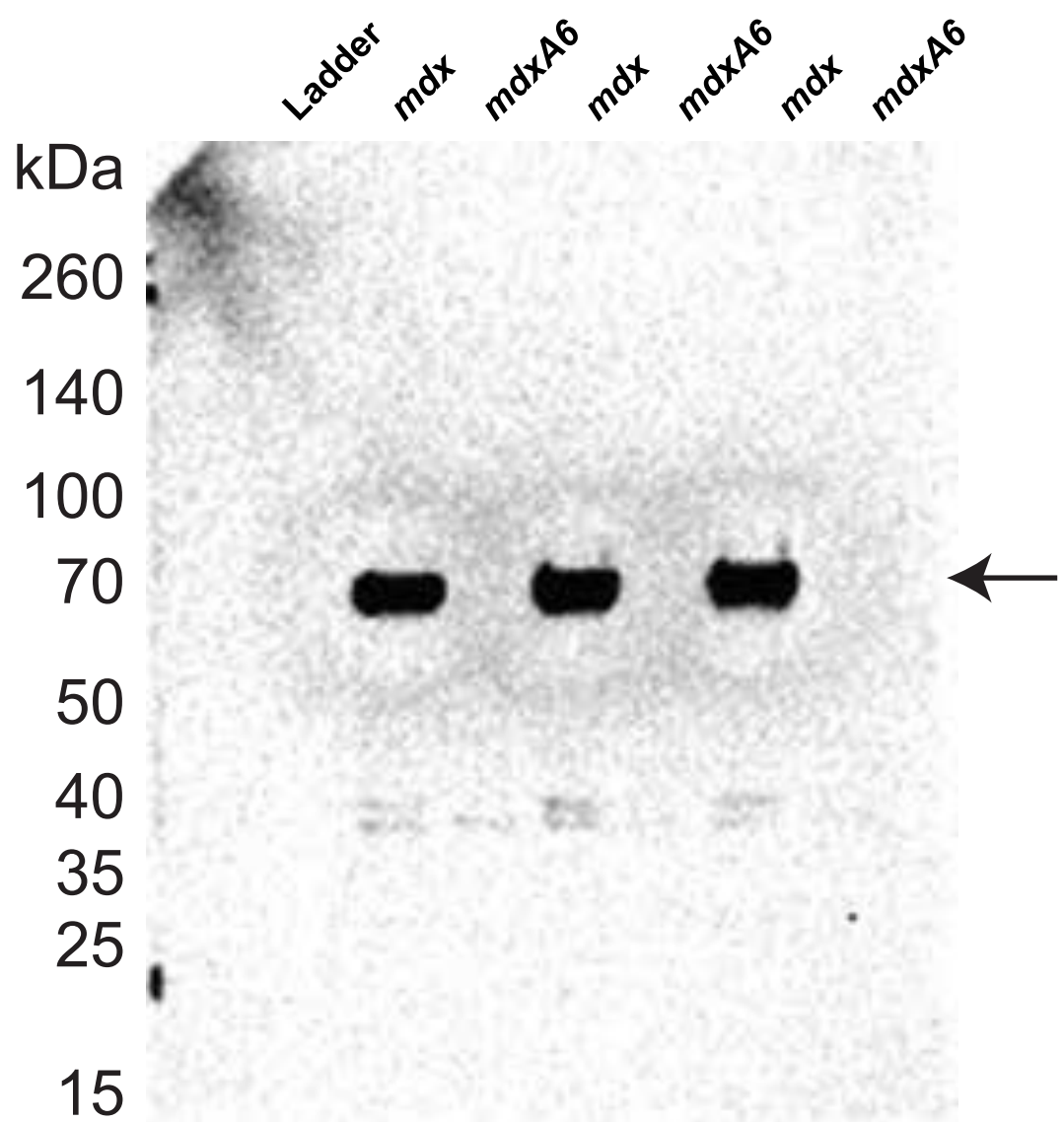

ANXA6

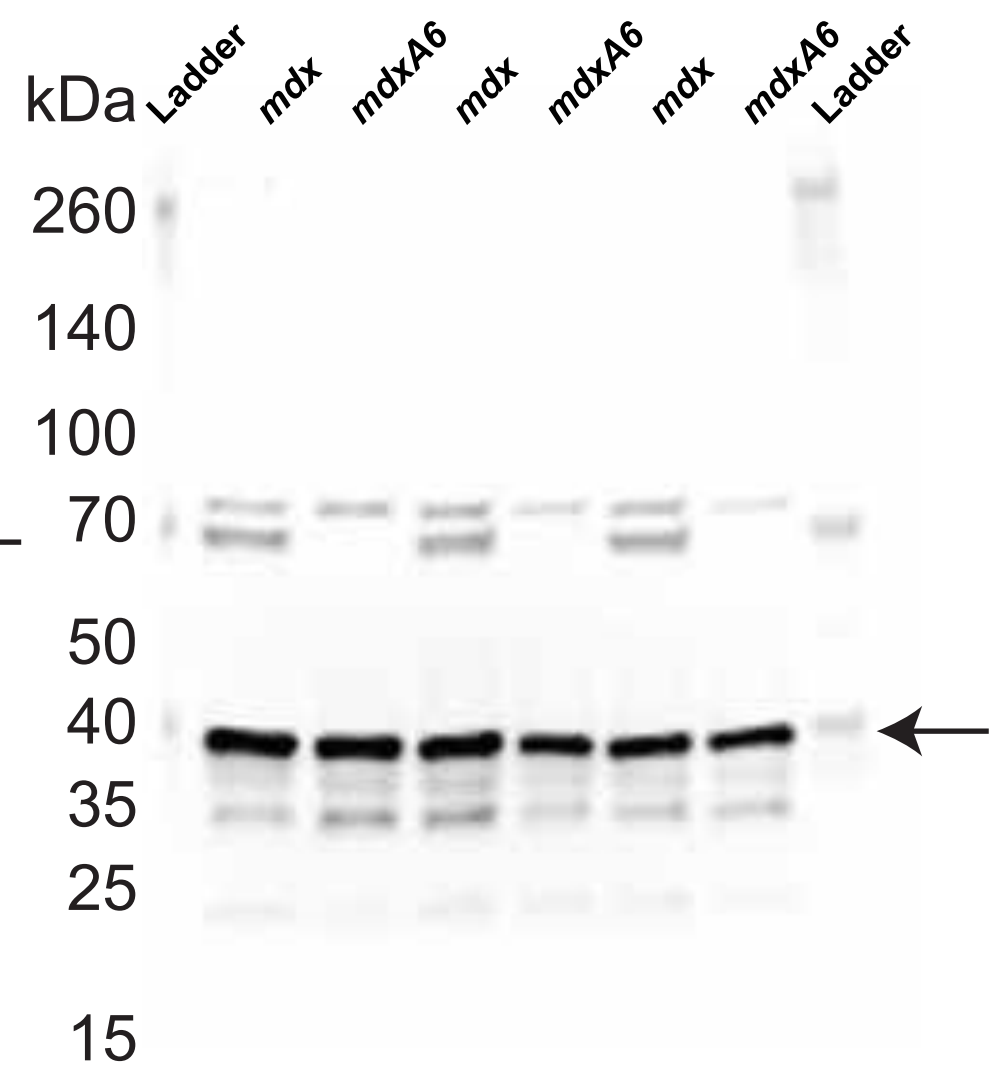

ANXA1

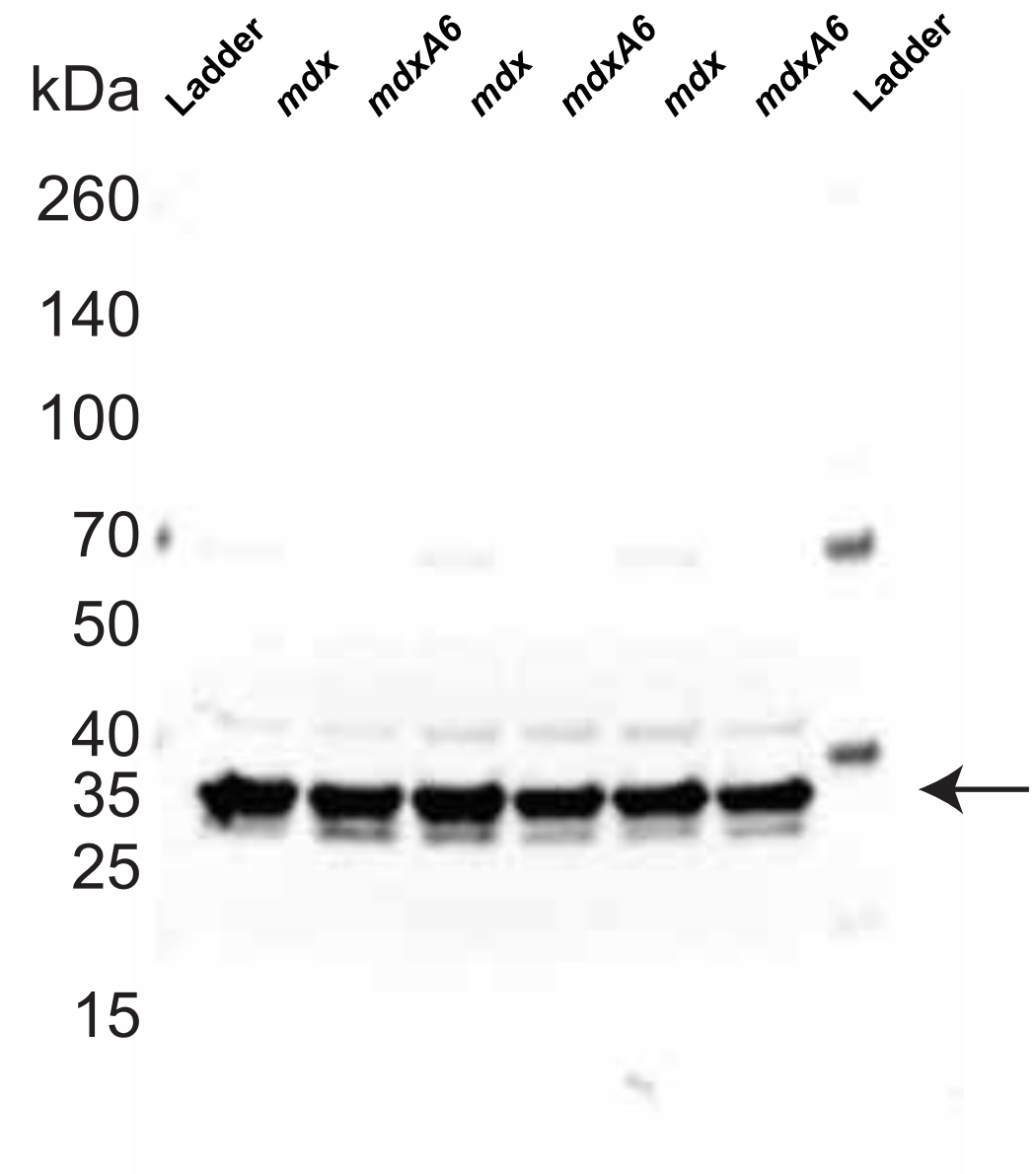

ANXA2

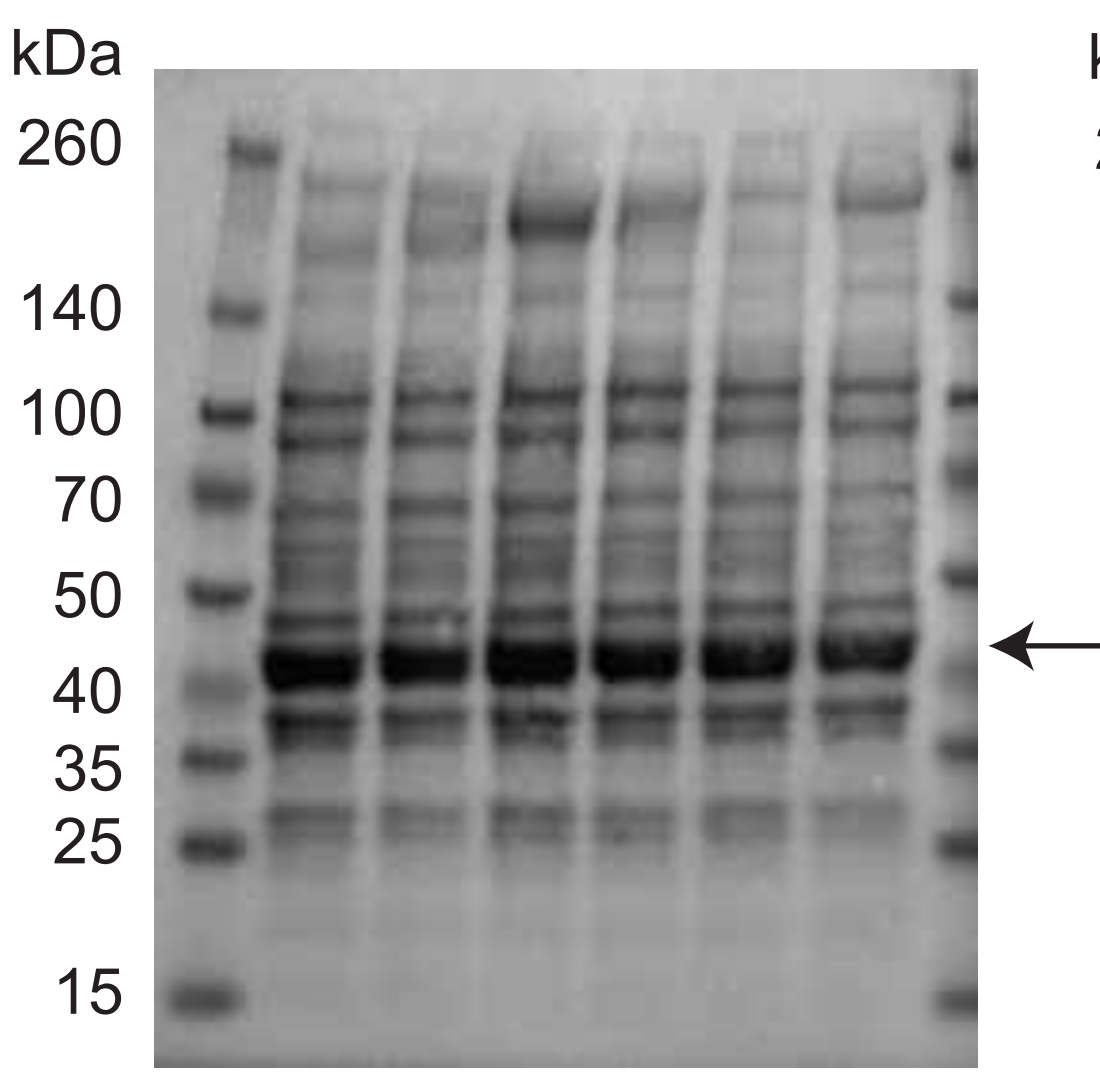

memcode

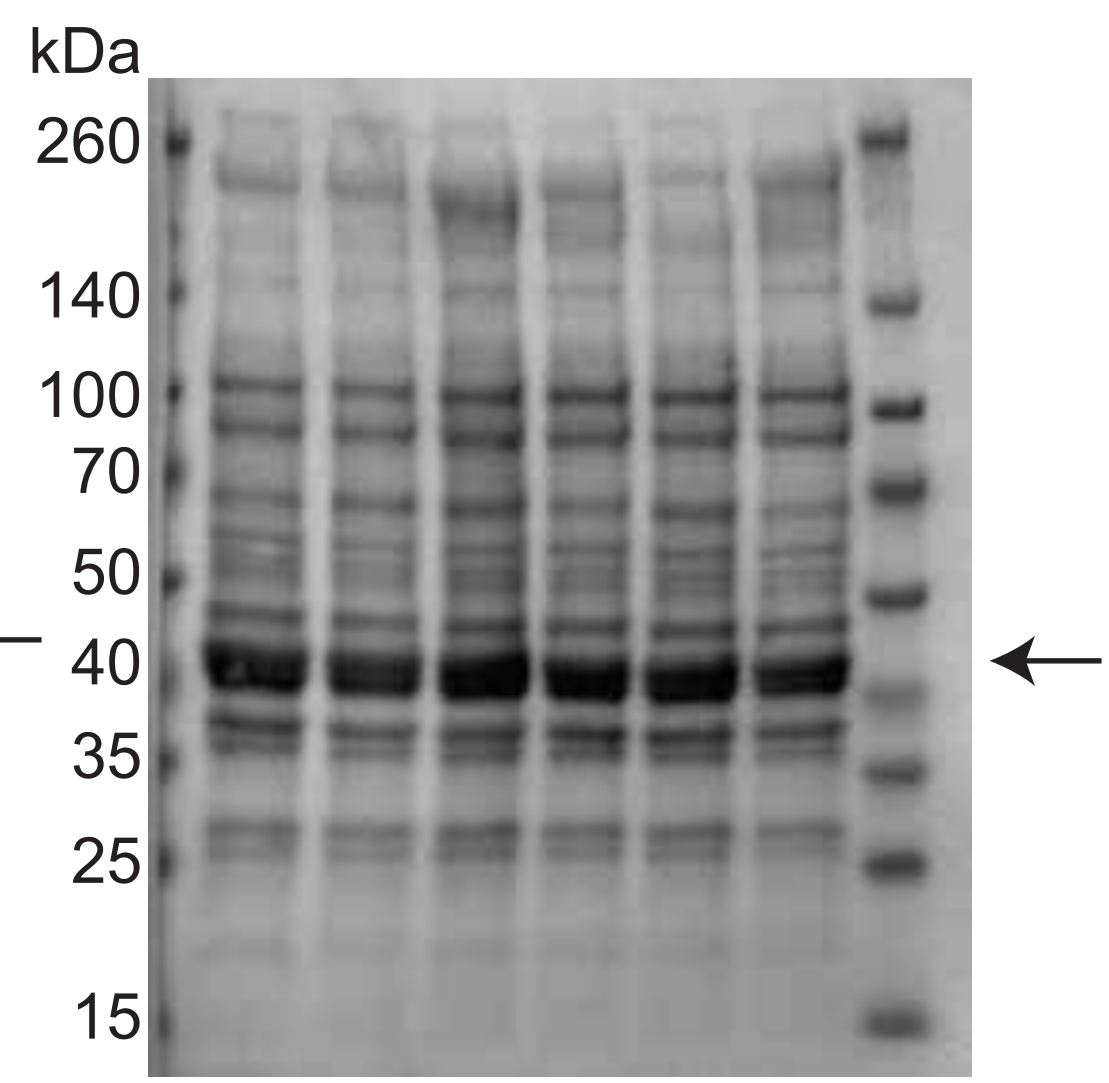

memcode

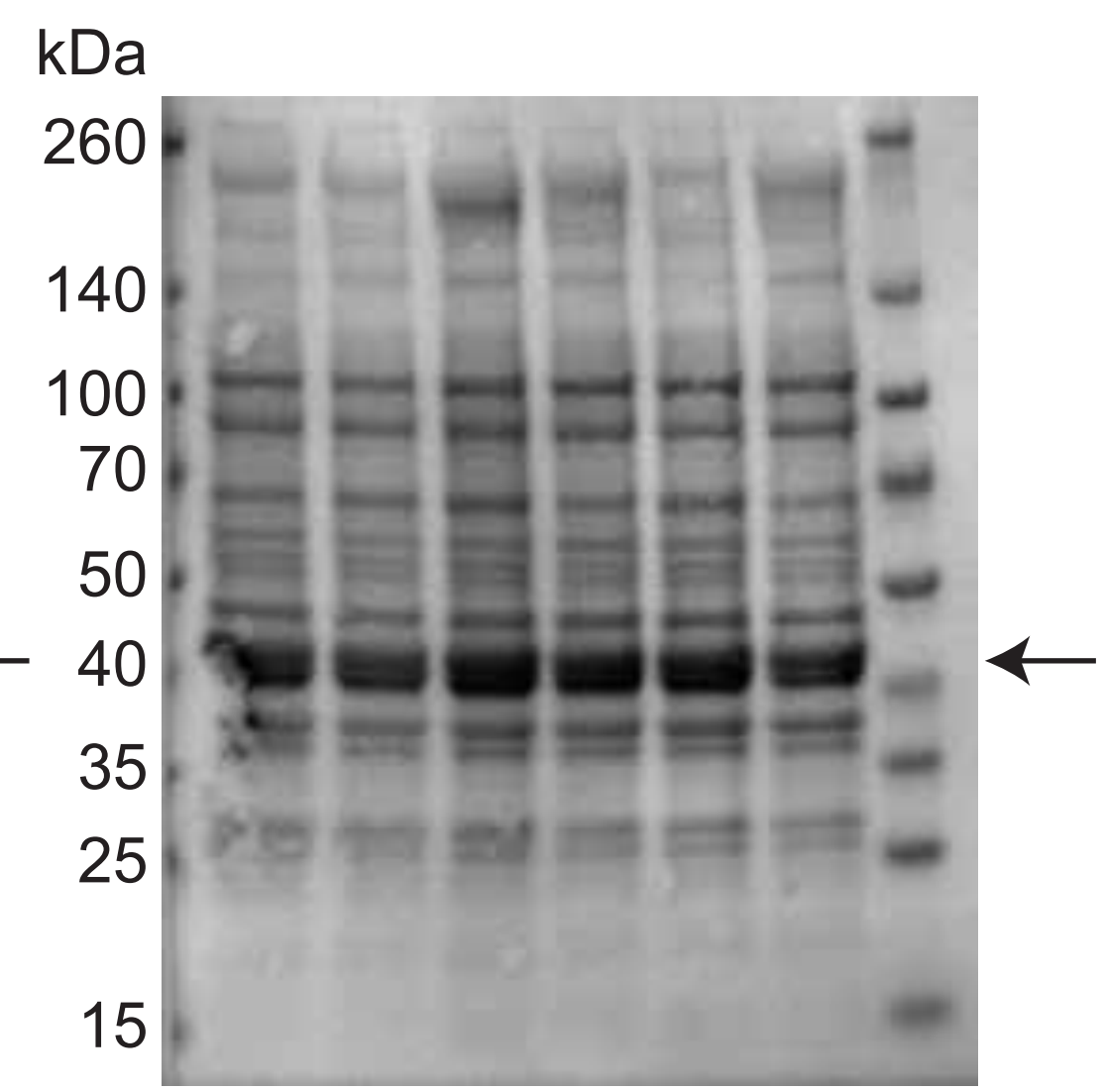

memcode
